# A meritorious integrated medical regimen for hepatic fibrosis and its complications *via* the systematic review and meta-analysis for Dahuang Zhechong pill-based therapy

**DOI:** 10.3389/fmed.2022.920062

**Published:** 2022-10-14

**Authors:** Zhen Ye, Qinfeng Huang, Yingqi She, Yu Hu, Mingquan Wu, Kaihua Qin, Linzhen Li, Chuantao Zhang, Xiaohong Zuo, Ailing Wei, Dewen Mao, Qiaobo Ye

**Affiliations:** ^1^School of Basic Medical Sciences, Chengdu University of Traditional Chinese Medicine, Chengdu, China; ^2^Department of Oncology, The First Affiliated Hospital, Guangxi University of Chinese Medicine, Nanning, China; ^3^Department of Pharmacy, Sichuan Orthopedic Hospital, Chengdu, Sichuan, China; ^4^Health Preservation and Rehabilitation College, Chengdu University of Traditional Chinese Medicine, Chengdu, Sichuan, China; ^5^Department of Respiratory Medicine, Hospital of Chengdu University of Traditional Chinese Medicine, Chengdu, Sichuan, China; ^6^Department of Liver Disease, The First Affiliated Hospital, Guangxi University of Chinese Medicine, Nanning, China

**Keywords:** Dahuang Zhechong pill, hepatic fibrosis, therapy, systematic review, meta-analysis

## Abstract

**Background:**

Hepatic fibrosis is a health challenge due to the absence of satisfactory therapy, especially at the cirrhosis stage. Dahuang Zhechong pill (DHZCP)-based therapy is reportedly a successful treatment for hepatic fibrosis and is even beneficial for the treatment of cirrhosis. Hence, a systematic review and clinical evidence assessment of DHZCP-based therapy should be performed, and clinical recommendations based on its efficacy for the treatment of hepatic fibrosis should be generated. With respect to potential indicators, the comparative value of the hepatic function, spleen thickness, and portal vein internal diameter should be evaluated.

**Materials and methods:**

PubMed, the Excerpta Medica Database, the Cochrane Library, the Web of Science, the WanFang Database, the Chinese Scientific Journal Database, and the Chinese National Knowledge Infrastructure database were searched to identify clinical trials. Three subgroup analyses were performed based on the stage of disease, medication use, and the course of treatment. Statistical analyses were performed using Review Manager 5.4.

**Results:**

A total of 18 studies including 1,494 patients were evaluated. The DHZCP-based therapy was effective in reducing the plasma levels of hyaluronic acid, and laminin, procollagen III, and IV collagen were also reduced irrespective of the hepatitis stage or the presence of hepatic cirrhosis. Abnormalities in alanine aminotransferase, aspartate aminotransferase, albumin, and total bilirubin were reversed. A 6-month course of treatment was the most beneficial DHZCP-based therapy regimen. Alanine aminotransferase improvement was more obvious in patients with cirrhosis, and alanine aminotransferase was reduced significantly in patients with hepatic cirrhosis. With respect to pharmacological mechanisms, DHZCP-based therapy could inhibit hepatic stellate cell growth and activation, reduce inflammation, and prevent extracellular matrix formation. Hepatic portal hypertension and splenomegaly were ameliorated significantly in the DHZCP-based therapy group.

**Conclusion:**

Dahuang Zhechong pill-based therapy has demonstrated efficacy as a treatment for hepatic fibrosis and cirrhosis. A 6-month course of treatment is the recommended option for DHZCP-based therapy in clinical practice. The combination of DHZCP-based therapy and entecavir is a favorable treatment for hepatic cirrhosis.

## Introduction

Hepatic fibrosis is caused by repeated injury healing and scar formation, which is a consequence of long-term persistent hepatocellular damage. Liver conditions, such as non-alcoholic fatty liver, alcoholic fatty liver, and viral hepatitis, can all lead to hepatic fibrosis, and of these, viral hepatitis has the highest prevalence. In recent years, chronic liver disease (CLD) has induced cirrhosis in approximately 633,000 patients per annum, with a global prevalence of 4.5–9.0% (1). Hepatocellular carcinoma (HCC) is the end stage of hepatic cirrhosis, the incidence of which is currently increasing worldwide. The global incidence of HCC exceeds 500,000 cases per year with the highest incidences in Asia and Africa, associated with the high prevalence of chronic hepatitis B (CHB) and chronic hepatitis C (CHC) in these regions (2, 3). Hepatic fibrosis, the end stage of which is hepatic cirrhosis, is an intermediate stage between malignant liver diseases and the early stage of hepatitis. This process needs to be effectively controlled and managed in the clinic. Based on this, the current urgent situation in patients with CLD has heightened the need to investigate therapies to improve hepatic fibrosis and the liver microenvironment.

Antiviral drugs can reverse the hepatic fibrosis process. Regrettably, alleviating pathogenic infections is a slow process during the reversal of hepatic fibrosis (4). The efficacy of this treatment is considered poor in patients with advanced hepatic cirrhosis, for which the only effective treatment is liver transplantation (5). The therapeutic effects of antiviral drugs on liver function and the liver microenvironment are unclear, as are their associated complications. The adverse effects of antiviral medications have raised public concerns, particularly with respect to long-term courses of treatment (6). This approach to treating hepatic fibrosis is limited; thus, there is a need to develop more effective drugs or treatment methods.

As an important and accessible medical resource, Chinese medicine has been indicated to increase the viral conversion rate, reverse hepatic fibrosis, improve liver microcirculation, and reduce adverse effects in CLD patients (7–10). Dahuang Zhechong pills (DHZ) (also known as “Rhubarb and Eupolyphaga Pills” or “Rhubarb and Ground Beetle Pills”), a classic Chinese medicine formula, were originally described in *Essentials from the Golden Cabinet*. The action of this medicinal formula include dispelling blood stasis and promoting new blood, and its original indications are severe deficiency due to exhaustion or lesions from the five labors: emaciation, fullness in the lower abdomen, loss of appetite, scaly skin, and dark eyes (11). DHZCP could be widely used to treat internal and external conditions, including gynecological diseases, silicosis, and prostate cancer, among others (11–14). Based on the theory of traditional Chinese medicine, blood stasis that stagnates in the liver can lead to liver diseases (11, 12). Therefore, it has been extensively used to treat various liver diseases, particularly hepatitis and hepatic fibrosis (11–14). Notably, DHZCP contains various active ingredients, such as anthraquinones, flavonoids, amino acids, and fatty acids. These components provide a material basis for its action. Pharmacological studies indicate that the effects of DHZCP for the treatment of hepatic fibrosis involve inhibiting inflammation, suppressing hepatic stellate cell (HSC) activation, downregulating fibronectin expression, and promoting microcirculation (15).

To date, the effects of DHZCP for CLD have been identified based on preclinical and clinical studies (15–18). The pharmacological effects of DHZCP for the treatment of CLD involve multiple targets and pathways, and antiviral activation. It has been suggested that combining DHZCP with conventional clinical treatment may be more beneficial than either treatment option alone, but whether DHZCP-based therapy combined with conventional liver-protective or antiviral drugs can effectively treat hepatic fibrosis or associated complications remains unclear. It is therefore necessary to analyze and evaluate the clinical application of DHZCP for the treatment of hepatic fibrosis further. The current meta-analysis systematically assessed clinical evidence pertaining to DHZCP combined with conventional liver-protective antiviral drugs for the treatment of hepatic fibrosis, and relevant aspects of the liver microenvironment, *via* evaluation of liver function, imaging indexes, and antiviral effects. A promising clinical protocol based on clinical evidence and pharmacological mechanisms of DHZCP is proposed. A meritorious integrated medical regimen is suggested for the treatment of hepatic fibrosis and associated complications, which could be an ideal treatment for hepatic cirrhosis.

## Materials and methods

The present study was conducted in accordance with the Preferred Reporting Items for Systematic Reviews and Meta-Analyses (PRISMA) statement (19). Every step was conducted by at least two authors separately. All discrepancies were resolved *via* discussion.

### Study selection

PubMed, the Excerpta Medica Database, the Cochrane Library, Web of Science, the WanFang Database, the Chinese Scientific Journal Database, and the Chinese National Knowledge Infrastructure database were searched independently. The terms used for searching are listed in the Supplementary material. No language or country restrictions were applied. Clinical trial registries^1^ and^2^ were also searched. Trials that met the requirements were considered. We also tried to contact the trial registrants by email to obtain appropriate raw data. Information pertaining to the studies included is presented in Table 1.

**TABLE 1:** The characteristics of included clinical trials.

First author (Year)	Course of treatment	Sample	Diagnosis	Stage	Drug duration (Intervention/Control)	Indexes
Cheng Menghuai (29)	24 weeks	31/30	CHB	Hepatic cirrhosis	DHZCP + Diammonium Glycyrrhizinate + Potassium magnesium aspartate + Reductive glutathione + Adefovir./Diammonium Glycyrrhizinate + Potassium magnesium aspartate + Reductive glutathione + Adefovir.	A, B, D, E, F, G, H;
Feng Xiaohong (28)	48 weeks	30/30	CHB	Hepatitis	DHZCP + Interferon-α./Interferon-α.	A, E;
Guo Jingjing (18)	48 weeks	59/60	CHB	Hepatitis	DHZCP **+** Tenofovir./Tenofovir.	A, B, C, D, E, F, H;
Dingchun et al. (61)	24 weeks	31/31	CHB	Hepatitis	DHZCP + Adefovir./Adefovir.	A, B, C, D, E, F, G, H;
Hong Hailong (21)	24 weeks	43/43	CHB	Hepatic cirrhosis	DHZCP + Entecavir./Entecavir.	E, F;
Liang Xiaoli (30)	3 months	30/30	CHB	Hepatic cirrhosis	DHZCP + Tenofovir./Tenofovir.	A, B, C, E, G, H;
Liu Weiwei (16)	24 weeks	34/33	CHB	Hepatitis	DHZCP + Entecavir + Diammonium Glycyrrhizinate./Entecavir + Diammonium Glycyrrhizinate.	A, B, C, D, E, F, G, H;
Liu Yubin (26)	24 weeks	25/25	CHB	Hepatic cirrhosis	DHZCP + Entecavir./Entecavir.	A, B, C, D, E, G, H;
Liu Xiaoyan (27)	48 weeks	66/62	CHB	Hepatic cirrhosis	DHZCP + Adefovir./Adefovir.	E, F, G, H;
Lin Jianhui (36)	3 months	38/38	CHB	Hepatitis	DHZCP + Reductive glutathione + Kuhuang./Reductive glutathione + Kuhuang.	A, B, D;
Miao Yongding (32)	6 months	42/36	CHB	Hepatitis	DHZCP + Lamivudine./Lamivudine.	A, B, C, D;
Pan Wen (19)	12 months	47/47	CHB	Hepatic cirrhosis	DHZCP + Entecavir + Protect liver treatment./Entecavir + Protect liver treatment.	A, E, G, H;
Shen Zhongliang (35)	3 months	43/39	CHB	Hepatitis	DHZCP + Glucurolactone + Legalon + Diammonium Glycyrrhizinate. /Glucurolactone + Legalon + Diammonium Glycyrrhizinate.	A, B, C, E;
Tian Guiqin (34)	2 months	56/56	CHB	Hepatitis	DHZCP + Protect liver treatment./Protect liver treatment.	A, B, C;
Xu Qian (33)	NA	45/45	CHB	Hepatitis	DHZCP + Legalon + Wuzhi capsules + Anethole trithine/Legalon + Wuzhi capsules + Anethole trithine.	A, B, D, E, H;
YAO Zhongcai (22)	24 weeks	32/32	CHB	Hepatic cirrhosis	DHZC Capsules** +** Entecavir./Entecavir.	A, B, C, D, E, F, H;
Zhang Hongxing (24)	6 months	32/32	CHB	Hepatic cirrhosis	DHZCP + Entecavir./Entecavir.	A, B, C, D, E, F, H;
Zhang Bangjie (31)	12 months	23/22	CHB	Hepatic cirrhosis	DHZCP + Adefovir/Adefovir	A, B, C, D;
Zhou Xiaolei (25)	48 weeks	48/48	CHB	Hepatitis	DHZCP + Entecavir./Entecavir.	A, B, D;

DHZCP, Dahuang Zhechong pill/capsule; CHB, chronic hepatitis B; CHC, chronic hepatitis C; NA, not applicable; A, HA; B, LN; C, PC III; D, IVC; E, ALT; F, AST; G, ALB; H, Tbil.

### Criteria for inclusion and exclusion

The inclusion and exclusion criteria were designed based on the principle of “Participants, Intervention, Control, Outcome, and Study Design” (PICOS). Only patients diagnosed with hepatic fibrosis or hepatic cirrhosis were included. Only studies reporting the results of DHZCP-based therapy combined with common clinical medicine, such as entecavir, lafamidine, adefovir, or interferon, were included. Studies involving acupuncture, surgery, other medicinal formulas, or Taiji were excluded. Studies in which the control treatment was other herbal formulas, acupuncture, or surgery were excluded. Hyaluronic acid (HA) was classified as a primary outcome that reflected hepatic fibrosis status. Laminin (LN), procollagen III (PCIII), IV collagen (IVC), alanine aminotransferase (ALT), aspartate aminotransferase (AST), albumin (ALB), total bilirubin (Tbil), spleen thickness, and portal vein internal diameter were included as secondary outcomes. ALT, AST, ALB, and Tbil reflect liver function. Reported data for at least one of these outcomes were required for study inclusion. Only clinical randomized controlled trials were included. Animal studies, reviews, meta-analyses, systematic evaluations, and case reports were excluded.

### Risk of bias assessments

The Cochrane Handbook was used to assess the risk of bias in the randomized controlled trials. Two reviewers analyzed the risk of bias independently. The items evaluated were (1) random sequence generation, (2) allocation concealment, (3) blinding of participants and personnel, (4) blinding of outcome assessment, (5) incomplete outcome data, (6) selective reporting, and (7) other biases. Each part was graded based on three levels of methodological quality: high risk, low risk, and unclear risk. Studies assessed as “high risk” were excluded.

### Statistical analysis

The meta-analysis was conducted using Review Manager 5.4. Analyses were performed using mean ± the standard deviation (M ± SD). Homogeneity was identified when *p* > 0.1 and *I*^2^ < 50%, and homogenous outcomes were used in a fixed-effects model. A random-effects model was used for heterogeneous outcomes. The mean difference (MD) values were used to measure the absolute difference in mean value between the two groups in this study. Weighted mean differences with 95% confidence intervals (CIs) were calculated for continuous data, and risk ratios (RRs) with 95% CIs were calculated for categorical data. A *P*-value < 0.05 was deemed to indicate statistical significance.

## Results

### Literature research

**FIGURE 1:**
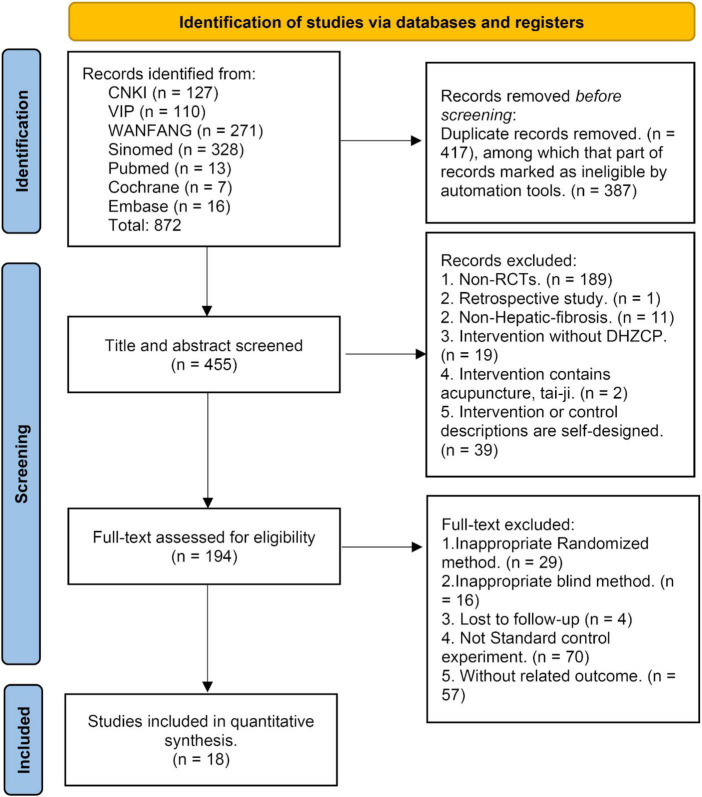
The literature research result [Preferred Reporting Items for Systematic Reviews and Meta-Analyses (PRISMA) flow diagram].

The initial database search yielded 872 studies. Of these, 417 duplicate studies were excluded before further screening. From the remaining 455 studies, 261 were excluded after the title and abstract screening. A further 176 studies were excluded after full-text screening based on PICOS principles. The remaining 18 studies were included in the meta-analysis (17, 18, 20–35), and they included collective totals of 755 patients in the experimental group and 739 patients in the control group (Figure 1).

### Study characteristics

A total of 1,494 participants were ultimately included in the analysis. The participants’ diseases included viral hepatitis B, viral hepatitis C, and hepatic cirrhosis. The interventions included DHZCP (or capsules of the same decoction) combined with adefovir, entecavir, glyburide, lavamidine, tenofovir, or interferon-alpha. The control group consisted of patients who underwent the same interventions without DHZCP or analogous capsules. Specific information on DHZCPs is presented in the Supplementary material (36–40).

### Risk of bias

**FIGURE 2:**
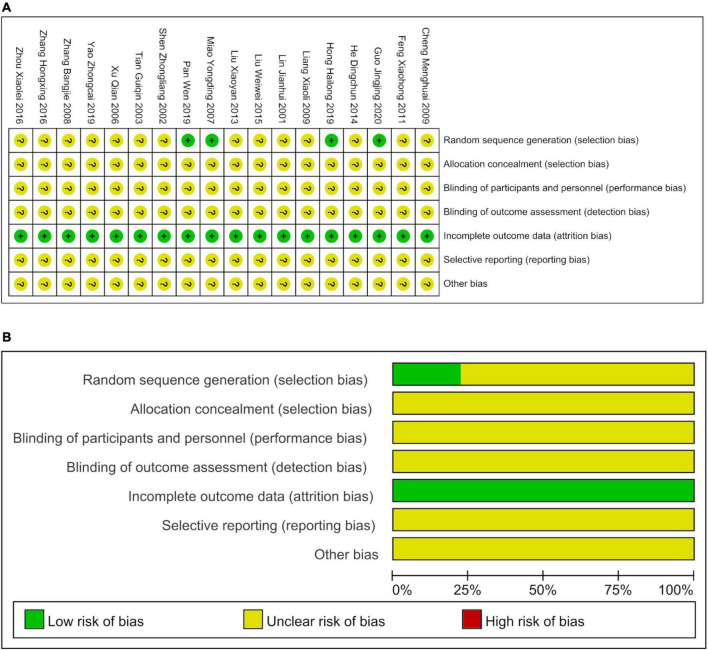
The bias risks of included studies. **(A)** The details of the bias risks. **(B)** The percentages of the risk of bias.

The 18 studies were evaluated using the Cochrane Risk of Bias Assessment Tool. The risk of bias due to the random assignment was low in four studies, while the specific random assignment method was unclear in the remaining 14 studies (Figure 2). Allocation concealment methods and double blinding methods were unclear in all 18 studies. No studies reported missed visits, and indicators of inadequate outcomes were low. Publication bias of primary outcomes was evident, as indicated in the Supplementary material. Other sources of bias were absent.

### Data analysis

#### Hyaluronic acid: A major serum marker of hepatic fibrosis

**FIGURE 3:**
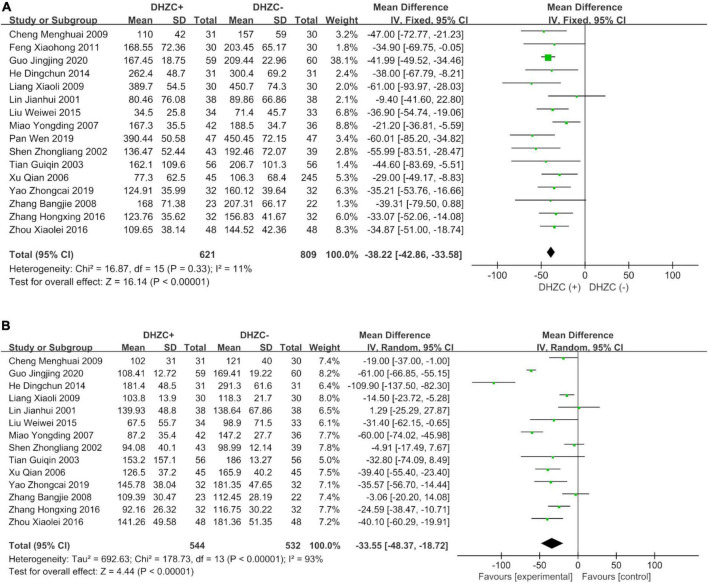
The effect of Dahuang Zhechong pill (DHZCP)-based therapy on hyaluronic acid (HA) and laminin (LN). Fixed-effects models were applied in panel **(A)**. Random-effects models were applied in panel **(B)**. **(A)** Total HA; **(B)** Total LN.

The HA level is considered as a specific serum indicator of hepatic fibrosis. The current analysis indicated that the DHZCP-based therapy significantly reversed the pathogenic upregulation of HA (*n* = 1,403; MD = −38.22; 95% CI −42.86 to −33.58; *p* < 0.00001; *I*^2^ = 11%) (Figure 3A). Three subgroup analyses of HA were performed (treatment course, disease stage, and medication use) with the aim of ascertaining the therapeutic effects of DHZCP-based therapy.

##### Course of treatment

The HA level was significantly decreased in the DHZCP-based therapy group after 3 months of treatment (*n* = 330; MD = −43.61; 95% CI −59.71 to −27.51; *p* < 0.00001, *I*^2^ = 51%), 6 months of treatment (*n* = 396; MD = −32.82; 95% CI −40.84 to −24.80; *p* < 0.00001, *I*^2^ = 0%), and 12 months of treatment (*n* = 354; MD = −41.96; 95% CI −48.46 to −35.47; *p* < 0.00001, *I*^2^ = 0%). Subgroup differences demonstrated that 3, 6, and 12 months are ideal courses of treatment (Supplementary Figure 1A).

##### Disease stage

The DHZCP-based therapy decreased the level of HA at the hepatitis stage (*n* = 843; MD = −37.66; 95% CI −42.95 to −32.36; *p* < 0.00001, *I*^2^ = 21%). This positive effect was also evident at the hepatic cirrhosis stage (*n* = 327; MD = −41.97; 95% CI −52.63 to −31.30; *p* < 0.00001, *I*^2^ = 13%). Subgroup differences were not significant (Supplementary Figure 1B).

##### Medication use

The results demonstrated that DHZCP combined with entecavir significantly reduced HA plasma levels compared to the control group (*n* = 224; MD = −34.45; 95% CI −44.70 to −24.20; *p* < 0.00001, *I*^2^ = 0%). DHZCP combined with adefovir significantly reduced HA plasma levels (*n* = 107; MD = −38.46; 95% CI −62.40 to −14.53; *p* = 0.002, *I*^2^ = 0%). DHZCP combined with other therapies significantly reduced the plasma levels of HA (*n* = 839; MD = −39.68; 95% CI −45.16 to −34.19; *p* < 0.00001, *I*^2^ = 43%) (Supplementary Figure 2A).

#### Laminin, procollagen III, and IV collagen: Other hepatic fibrosis serum markers

**FIGURE 4:**
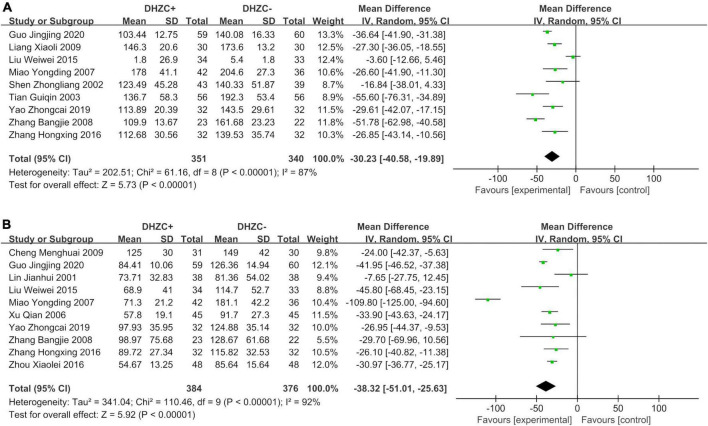
The effect of Dahuang Zhechong pill (DHZCP)-based therapy on procollagen III (PCIII) and IV collagen (IVC). Random-effects models were applied. **(A)** Total PCIII; **(B)** Total IVC.

In terms of total effect, the DHZCP-based therapy group exhibited significant decreases in the levels of LN (*n* = 1076; MD = −33.55; 95% CI −48.37 to −18.72; *p* < 0.00001, *I*^2^ = 93%; Figure 3B), PCIII (*n* = 691; MD = −30.23; 95% CI −40.58 to −19.89; *p* < 0.00001, *I*^2^ = 87%; Figure 4A), and IVC (*n* = 760; MD = −38.32; 95% CI −51.01 to −25.63; *p* < 0.00001, *I*^2^ = 92%; Figure 4B).

##### Course of treatment

The LN level was significantly reduced in the DHZCP-based therapy group after 3 months of treatment (*n* = 330; MD = −10.61; 95% CI −18.56 to −2.66; *p* = 0.009, *I*^2^ = 10%) and 6 months of treatment (*n* = 396; MD = −45.81; 95% CI −68.66 to −22.95; *p* < 0.0001, *I*^2^ = 88%). There was no statistically significant difference in the LN levels between the DHZCP-based therapy group and the control group after 12 months of treatment (*n* = 260; MD = −35.25; 95% CI −72.11 to 1.60; *p* = 0.06, *I*^2^ = 95%) (Supplementary Figure 2B).

The PCIII level was significantly reduced in the DHZCP-based therapy group after 3 months of treatment (*n* = 254; MD = −32.60; 95% CI −51.45 to −13.76; *p* = 0.0007, *I*^2^ = 74%), 6 months of treatment (*n* = 195; MD = −9.37; 95% CI −37.81 to −0.92; *p* = 0.04, *I*^2^ = 85%), and 12 months of treatment (*n* = 164; MD = −43.37; 95% CI −58.11 to −28.62; *p* < 0.00001, *I*^2^ = 83%) (Supplementary Figure 3A).

The IVC level was significantly reduced in the DHZCP-based therapy group after 6 months of treatment (*n* = 256; MD = −28.83; 95% CI −37.66 to −20.00; *p* < 0.00001, *I*^2^ = 0%). IVC was significantly lower in the DHZCP-based therapy group than in the control group after 12 months of treatment (*n* = 260; MD = −36.26; 95% CI −46.08 to −26.44; *p* < 0.00001, *I*^2^ = 77%) (Supplementary Figure 3B).

##### Disease stage

The LN level at the hepatitis stage was significantly better in the DHZCP-based therapy group than in the control group (*n* = 782; MD = −1.12; 95% CI −1.79 to −0.44; *p* = 0.001, *I*^2^ = 95%), and there was also a significant improvement at the cirrhotic stage (*n* = 294; MD = −0.64; 95% CI −0.90 to −0.38; *p* < 0.00001, *I*^2^ = 18%) (Supplementary Figure 4A). PCIII at the hepatitis stage was significantly better in the DHZCP-based therapy group than in the control group (*n* = 470; MD = −29.69; 95% CI −47.42 to −11.96; *p* = 0.001, *I*^2^ = 92%). There was also a significant improvement at the cirrhotic stage (*n* = 233; MD = −34.14; 95% CI −46.49 to −21.78; *p* < 0.00001, *I*^2^ = 77%) (Supplementary Figure 4B). IVC at the hepatitis stage was significantly better in the DHZCP-based therapy group than in the control group (*n* = 448; MD = −33.53; 95% CI −42.21 to −24.85; *p* < 0.00001, *I*^2^ = 78%). There was also a significant improvement at the cirrhotic stage (*n* = 234; MD = −26.00; 95% CI −35.32 to −16.67; *p* < 0.00001, *I*^2^ = 0%) (Supplementary Figure 5A).

##### Medication use

The use of DHZCP in combination with entecavir reduced the LN plasma levels significantly more than entecavir alone (*n* = 224; MD = −30.93; 95% CI −40.99 to −20.87; *p* < 0.00001, *I*^2^ = 0%). There was no statistically significant difference between DHZCP combined with adefovir and the control group (*n* = 107; MD = −55.91; 95% CI −160.61 to 48.79; *p* = 0.30, *I*^2^ = 98%). DHZCP in combination with other therapies significantly reduced serum LN levels (*n* = 745; MD = −29.55; 95% CI −48.29 to −10.81; *p* = 0.002, *I*^2^ = 94%) (Supplementary Figure 5B).

The use of DHZCP in combination with entecavir reduced PCIII serum levels significantly more than entecavir alone (*n* = 128; MD = −28.59; 95% CI −38.49 to −18.70; *p* < 0.00001, *I*^2^ = 0%). Only one study investigated the combination of DHZCP and adefovir, and therefore, the results should be interpreted with caution (*n* = 45; MD = −51.78; 95% CI −62.98 to −40.58; *p* < 0.00001). DHZCP combined with other therapies, such as conventional liver protection, significantly improved serum PCIII levels (*n* = 530; MD = −29.06; 95% CI −42.52 to −15.61; *p* < 0.0001, *I*^2^ = 90%) (Supplementary Figure 6A).

The use of DHZCP in combination with entecavir reduced IVC serum levels significantly more than entecavir alone (*n* = 224; MD = −30.02; 95% CI −35.18 to −24.87; *p* < 0.00001, *I*^2^ = 0%). Adefovir has only one data, and the results should be interpreted with caution (*n* = 45; MD = −29.70; 95% CI −69.96 to −10.56; *p* < 0.15). DHZCP combined with other therapies, such as conventional liver protection, significantly improved serum PCIII levels (*n* = 213; MD = −32.26; 95% CI −43.13 to −21.39; *p* < 0.00001, *I*^2^ = 73%) (Supplementary Figure 6B).

#### Liver function

**FIGURE 5:**
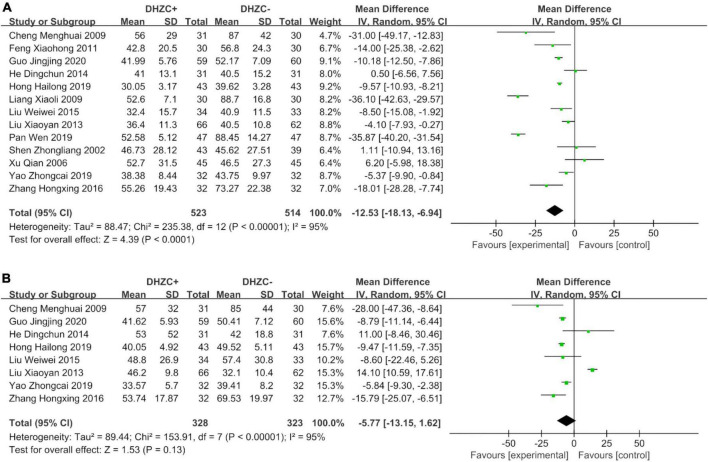
The effect of Dahuang Zhechong pill (DHZCP)-based therapy on alanine aminotransferase (ALT) and aspartate aminotransferase (AST). Random-effects models were applied. **(A)** Total ALT; **(B)** Total AST.

**FIGURE 6:**
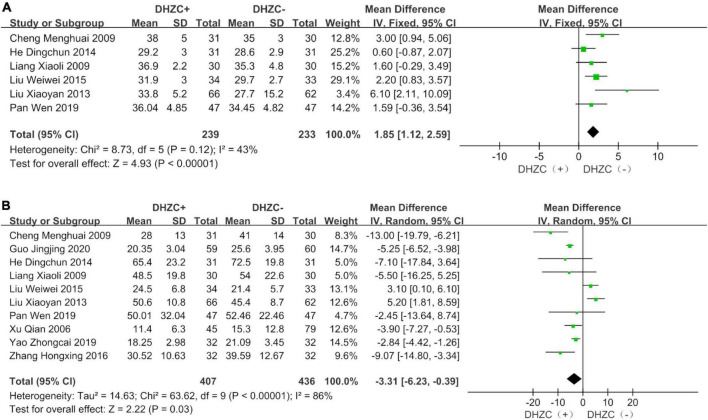
The effect of Dahuang Zhechong pill (DHZCP)-based therapy on albumin (ALB) and total bilirubin (Tbil). Fixed-effects models were applied in panel **(A)**. Random-effects models were applied in panel **(B)**. **(A)** Total ALB; **(B)** Total Tbil.

The DHZCP based-therapy group exhibited significant reductions in the levels of ALT (*n* = 1037; MD = −12.53; 95% CI −28.28 to −7.74; *p* < 0.0001, *I*^2^ = 95%; Figure 5A), AST (*n* = 651; MD = −5.77; 95% CI −13.15 to −1.62; *p* < 0.00001, *I*^2^ = 95%; Figure 5B), ALB (*n* = 472; MD = 1.85; 95% CI 1.12, 2.59; *p* = 0.03, *I*^2^ = 43%; Figure 6A), and Tbil (*n* = 843; MD = −3.31; 95% CI −6.23 to −0.39; *p* = 0.03, *I*^2^ = 86%; Figure 6B).

##### Course of treatment

Compared with the control group, the DHZCP-based therapy group exhibited a significant reduction in ALT after a 6-month course of treatment (*n* = 404; MD = −8.84; 95% CI −13.37 to −4.32; *p* = 0.0001, *I*^2^ = 73%). ALT was significantly reduced in the DHZCP-based therapy group after a 12-month course of treatment (*n* = 401; MD = −16.06; 95% CI −29.82 to −2.30; *p* = 0.02, *I*^2^ = 98%). After 3 months of treatment, there was no significant reduction in ALT in the experimental group (*n* = 142; MD = −17.85; 95% CI −54.31 to −18.60; *p* = 0.34, *I*^2^ = 96%) (Supplementary Figure 7A).

Compared with the control group, the DHZCP-based therapy group exhibited a significant reduction in AST after a 6-month course of treatment (*n* = 340; MD = −10.67; 95% CI −17.95 to −3.40; *p* = 0.004, *I*^2^ = 58%). There was no significant reduction in AST in the DHZCP-based therapy group after a 12-month course of treatment (*n* = 311; MD = −0.21; 95% CI −13.80 to 13.37; *p* = 0.98) (Supplementary Figure 7B).

Compared with the control group, the DHZCP-based therapy group exhibited a mild increase in ALB after a 6-month course of treatment (*n* = 190; MD = 1.82; 95% CI 0.49, 3.16; *p* = 0.007, *I*^2^ = 52%). There was no significant difference in ALB between the DHZCP-based therapy group and the control group after a 12-month course of treatment (*n* = 222; MD = 3.50; 95% CI −0.87 to 7.86; *p* = 0.12) (Supplementary Figure 8A). There were no significant differences in Tbil between the DHZCP-based therapy group and the control group after 6 months of treatment (*n* = 318; MD = −4.94; 95% CI −9.89 to 0.02; *p* = 0.05, *I*^2^ = 86%) or 12 months of treatment (*n* = 341; MD = −0.70; 95% CI −9.16 to 7.76; *p* = 0.87, *I*^2^ = 94%) (Supplementary Figure 8B).

##### Disease stage

**FIGURE 7:**
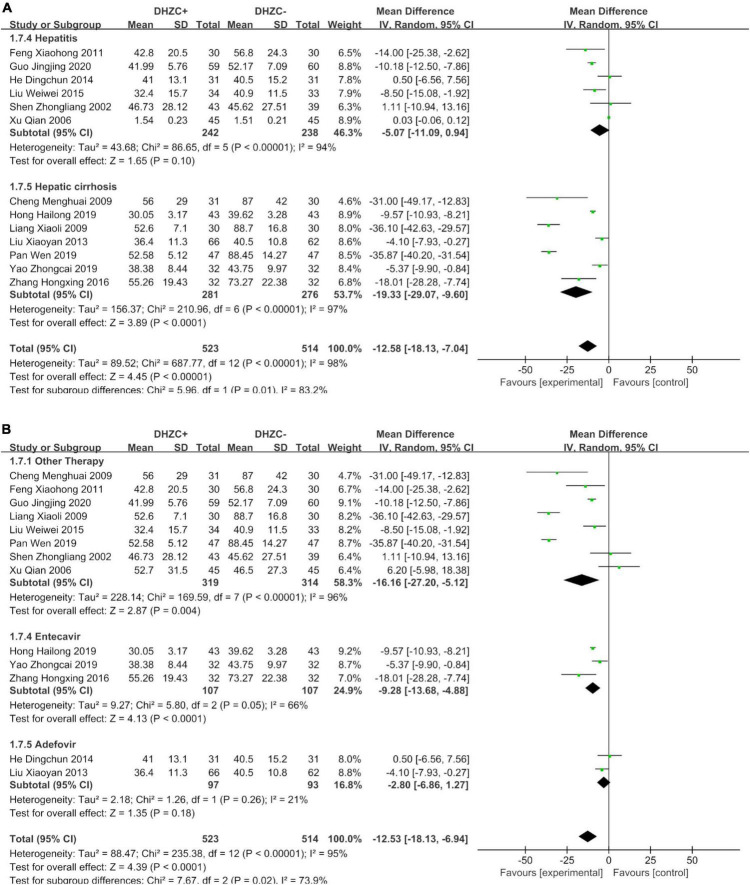
The effect of Dahuang Zhechong pill (DHZCP)-based therapy on alanine aminotransferase (ALT) with a subgroup of the stage of disease and medication use. Random-effects models were applied. **(A)** ALT with a subgroup of the stage of disease; **(B)** ALT with a subgroup of medication use.

Compared with the control group, there was a significantly greater reduction in ALT at the cirrhotic stage in the DHZCP-based therapy group (*n* = 557; MD = −19.33; 95% CI −29.07 to −9.60; *p* < 0.0001, *I*^2^ = 97%). There was no significant difference in ALT at the hepatitis stage between the DHZCP-based therapy group and the control group (*n* = 480; MD = −5.07; 95% CI −11.09 to 0.94; *p* = 0.10, *I*^2^ = 94%). For this index, the DHZCP-based therapy group exhibited an advantage at the cirrhotic stage (test for subgroup differences; Chi^2^ = 5.96, df = 1; *p* = 0.01, *I*^2^ = 83.2%) (Figure 7A).

The AST levels in the DHZCP-based therapy group did not differ significantly from those in the control group at the hepatitis stage (*n* = 248; MD = −5.66; 95% CI −14.53 to 3.21; *p* = 0.21, *I*^2^ = 49%) or the cirrhotic stage (*n* = 403; MD = −7.20; 95% CI −18.47 to 4.07; *p* = 0.21, *I*^2^ = 97%) (Supplementary Figure 9A). Compared to the control group, the DHZCP-based therapy group exhibited significant advantages with respect to elevated ALB levels at the hepatitis stage (*n* = 129; MD = 1.46; 95% CI 0.46 to 2.46; *p* = 0.004, *I*^2^ = 59%) and at the cirrhotic stage (*n* = 343; MD = 2.33; 95% CI 1.24 to 3.42; *p* < 0.0001, *I*^2^ = 40%) (Supplementary Figure 9B). There were no significant differences in Tbil between the DHZCP-based therapy group and the control group at the hepatitis stage (*n* = 338; MD = −2.01; 95% CI −6.65 to 2.64; *p* = 0.40, *I*^2^ = 89%) or the cirrhotic stage (*n* = 471; MD = −4.21; 95% CI −9.38 to 0.97; *p* = 0.11, *I*^2^ = 86%) (Supplementary Figure 10A).

##### Medication use

Compared with the control group, the ALT levels were significantly lower in patients treated with DHZCP combined with conventional liver protection (*n* = 633; MD = −16.16; 95% CI −27.20 to −5.12; *p* = 0.004, *I*^2^ = 96%) and in patients treated with DHZCP combined with entecavir (*n* = 214; MD = −9.28; 95% CI −13.68 to −4.88; *p* < 0.0001, *I*^2^ = 66%) but not in patients treated with DHZCP combined with adefovir (*n* = 190; MD = −12.53; 95% CI −18.13 to −6.94; *p* < 0.0001, *I*^2^ = 95%). This indicated that DHZCP combined with conventional liver protection or entecavir was better than DHZCP combined with adefovir (Figure 7B).

**FIGURE 8:**
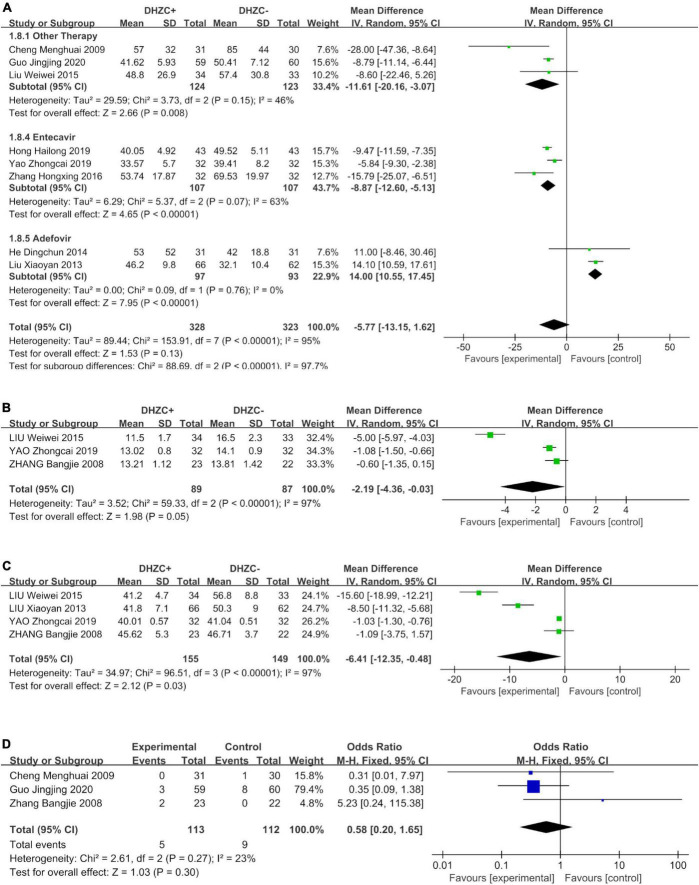
The effect of Dahuang Zhechong pill (DHZCP)-based therapy on albumin (ALB) with different courses of treatment, portal vein internal diameter, and splenic thickness. The adverse effects are also exhibited. Random-effects models were applied in panels **(A–C)**. Fixed-effects model was applied in panel **(D)**. **(A)** ALB with different courses of treatment; **(B)** Portal vein internal diameter; **(C)** Spleen thickness; and **(D)** Adverse effect.

Compared to the control group, DHZCP combined with entecavir was significantly advantageous with respect to reducing the AST levels (*n* = 214; MD = −8.87; 95% CI −12.60 to −5.13; *p* < 0.00001, *I*^2^ = 63%). The AST levels also differed significantly in patients who underwent DHZCP-based therapy combined with conventional liver protection and other treatments compared to the control group (*n* = 247; MD = −11.61; 95% CI −20.16 to −3.07; *p* = 0.008, *I*^2^ = 46%). Importantly, DHZCP combined with adefovir treatment significantly elevated AST (*n* = 190; MD = 14.00; 95% CI 10.55 to 17.45; *p* < 0.00001, *I*^2^ = 0%), indicating a potential toxic effect (test for subgroup differences: Chi^2^ = 88.69, df = 2; *p* < 0.00001, *I*^2^ = 97.7%) (Figure 8A).

Compared with the control group, DHZCP combined with other therapeutic treatment regimens resulted in significantly elevated ALB levels (*n* = 282; MD = 2.09; 95% CI 1.22 to 2.97; *p* < 0.00001, *I*^2^ = 0%). There was no significant difference in improvement in ALB between DHZCP combined with adefovir and the control group (*n* = 190; MD = 1.26; 95% CI −0.12 to 2.64; *p* = 0.07, *I*^2^ = 84%) (Supplementary Figure 10B).

There were no significant reductions in Tbil in patients treated with DHZCP combined with other therapies (*n* = 491; MD = −3.53; 95% CI −7.71 to 0.64; *p* = 0.01, *I*^2^ = 86%), DHZCP combined with entecavir (*n* = 128; MD = −5.32; 95% CI −11.30 to 0.66; *p* = 0.08, *I*^2^ = 76%), or DHZCP combined with adefovir (*n* = 190; MD = 0.15; 95% CI −11.71 to 12.01; *p* = 0.98, *I*^2^ = 78%) (Supplementary Figure 11).

#### Portal vein internal diameter and spleen thickness

Compared with the control group, the DHZCP-based therapy mildly reversed portal vein internal diameter widening (*n* = 176; MD = −2.19; 95% CI −4.36 to −0.03; *p* = 0.05, *I*^2^ = 97%, Figure 8B). There was also significant improvement in spleen thickness in the DHZCP group (*n* = 304; MD = −6.41; 95% CI −12.35 to −0.48; *p* = 0.03, *I*^2^ = 97%, Figure 8C). These results suggest that the DHZCP-based therapy can improve hepatic portal hypertension and reduce portal vein internal diameter while modulating local immune responses, suppressing local inflammation, and improving hypersplenism. However, the results need to be interpreted with caution due to high heterogeneity.

#### Adverse events

Three studies reported adverse events (Figure 8D). Those reported in the DHZCP-based therapy group included one case of abdominal pain with mild diarrhea, one case of headache, one case of dizziness, one case of nausea, and one case of generalized weakness. Those reported in the control group included three cases of dizziness, one case of rash, two cases of general weakness, two cases of nausea, and one case of mild alopecia. The number of adverse events did not differ significantly between the two groups (*n* = 225; MD = 0.58; 95% CI 0.20 to 1.65; *p* = 0.27, *I*^2^ = 23%).

## Discussion

Hepatic fibrosis is widely recognized as a crucial stage before malignant liver diseases. Evidence suggests that chronic viral hepatitis is the most critical factor in hepatic fibrosis development (5). DHZCP, a classic formula created by Zhang Zhong-jing, reportedly has the potential to treat hepatic fibrosis (15, 17, 18, 20–35). The formula consists of *Dà Huáng* (dried root and rhizome of *Rheum officinale* Baill.), *Méng Chóng* (dried *Tabanus*), *Shui Zhì* (dried *Whitmania pigra* Whitman.), *Tǔ Bi$\bar{e}$ Chóng* (dried *Eupolyphaga sinensis* Walker.), *Qí Cáo* (dried *Holotrichia*), *G$\bar{a}$n q$\bar{i}$* [dried *Toxicodendron vernicifluum* (Stokes) F. A. Barkley], *Xìng Rén* (dried seed of *Prunus armeniaca* L.), *Sháo Yào* (dried root of *Paeonia lactiflora* Pal.), *G$\bar{a}$n Cǎo* (dried root of *Glycyrrhiza uralensis* Fisch.), *Huáng Qín* (dried root of *Scutellaria baicalensis* Georgi.), *Táo Rén* [dried seed of *Prunus persica* (L.) Batsch], and *Sh$\bar{e}$ng Dì Huáng* (dried root of *Rehjnannia glutinosa* Libosch.) (11). To prepare the formula, the medicinals are ground into a fine powder, and then, honey is added to make pills (3 g for each pill) (11, 12, 36). The details of the preparation technics could be found in the Supplementary material.

Pharmacological studies indicate that DHZCP can have positive effects on the processes of hepatitis and hepatic fibrosis (15). Clinical studies showed that DHZCP has antifibrotic and anti-platelet agglutination effects, which positively affect liver cirrhosis. Wei et al. reported that DHZCP reduced liver fibrosis serum biomarkers in patients with chronic hepatitis B. Notably, however, little attention has been paid to the effects of DHZCP on liver function and hepatic fibrosis complications or the optimal course of treatment and duration of drug administration. The optimal DHZCP-based therapy regimen remains unclear (41). The current meta-analysis systematically reviewed and analyzed the clinical effects of DHZCP combined with conventional treatments for hepatic fibrosis and its complications. Various benefits of DHZCP-based therapy in the early stages of fibrosis and hepatic cirrhosis have been identified, but the appropriate course and the recommended medication window require further investigation.

Eighteen studies were included in the current meta-analysis, and sensitive indicators of hepatic fibrosis (HA, LN, PCIII, and IVC), liver function (ALT, AST, Tbil, and ALB), and complications (spleen thickness and portal vein internal diameter) (39) were evaluated. Subgroup analyses were performed to investigate different courses of treatment, stages of the disease, and concordantly administered medications. The course of treatment subgroups included 3, 6, and 12 months. All three durations of treatment significantly improved HA, PCIII, and IVC levels, whereas the effects of 12 months of treatment on LN were not clear. Liver function indicators were significantly improved after 6 months of treatment with DHZCP combined with conventional therapy. DHZCP combined with conventional therapy could effectively reduce hepatic fibrosis indexes at the hepatitis and cirrhosis stages, as well as ALB. However, ALT was only significantly reduced at the hepatic cirrhosis stage. Notably, DHZCP-based therapy was not associated with any significant improvement in AST or Tbil indexes in patients with hepatitis or hepatic cirrhosis. ALT and AST mainly exist in hepatocytes. The plasma levels of these aminotransferases are low under normal conditions. They infiltrate the blood when hepatocytes are damaged and cell permeability increases. In the current meta-analysis, DHZCP-based therapy resulted in a significant decrease in ALT, but no substantial changes in AST. DHZCP-based therapy effectively upregulated ALB and improved liver function, which also contributed to the healing of the liver injury. Additionally, the results indicated that DHZCP combined with entecavir is an effective treatment for hepatic fibrosis, and even hepatic cirrhosis. The combination of DHZCP and adefovir had several negative effects, however, indicating that this combination should be used cautiously in the clinic and that the combination of DHZCP and entecavir may be more suitable for treating hepatic fibrosis.

Hepatic fibrosis leads to cirrhosis, which in turn increases the burden on the portal vein, and can even lead to the generation of portal vein thrombosis. This condition often responds clinically to the widening of the internal diameter of the portal vein (42). An increase in spleen size is also evident during the development of fibrosis. Because the liver and spleen are closely related through the portal vein system, splenic immune cells and cytokines may influence the hepatic immune microenvironment *via* portal vein flow, and splenic immune cell dysregulation during hepatic cirrhosis may be caused by liver-derived damage-associated molecular patterns (DAMPs) or exons. Thus, many scholars consider spleen volume an essential indicator when assessing the severity of hepatic fibrosis (16). In the present study, increased portal vein internal diameter and splenic thickening caused by hepatic fibrosis were also evaluated. DHZCP-based therapy significantly reduced the inner diameter of the portal vein and spleen thickness, indicating that DHZCP can reverse compensatory widening of the portal vein and hypersplenism caused by hepatic fibrosis.

**FIGURE 9:**
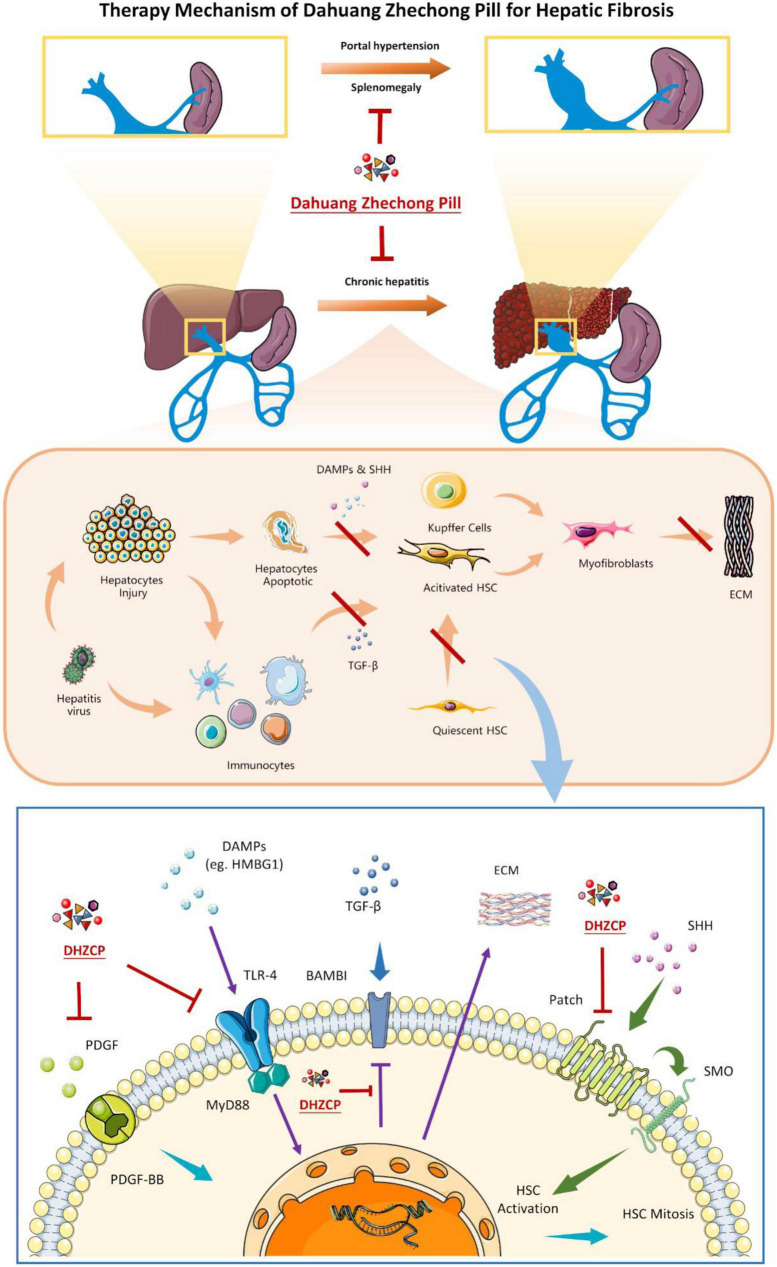
The metabolism of Dahuang Zhechong pill (DHZCP) in treating hepatic fibrosis according to the reported studies. In the molecular mechanism diagram **(the lowest picture)**, arrows with the same color indicate the same signaling pathway.

Pharmacological studies suggest that DHZCP can improve the pathological status of hepatitis and hepatic fibrosis by interfering with multiple molecular targets and blocking the abnormally activated signaling pathway. Excessive accumulation of extracellular matrix is one of the characteristics of hepatic fibrosis and is associated with excessive proliferation and activation of HSC and Kupffer cells (Figure 9). It has been suggested that HSCs can be used as specific targets for the treatment of hepatic fibrosis (43). DHZCP can inhibit abnormal activation of the hedgehog signaling pathway by downregulating the PATCH protein, thus inhibiting the activation of HSC. Platelet-derived growth factor (PDGF) is a potent HSC mitogen. DHZCP can reduce the expression of PDGF-BB and PDGF receptors, thus inhibiting HSC proliferation. The death of injured hepatocytes can result in the release of DAMPs. DAMPs can promote the proliferation and activation of HSCs and Kupffer cells into myofibroblasts, producing extracellular matrix and generating fibrous scarring (42, 44). In cases of short-term injury, this process can be balanced by the mechanism of scar dissolution, but an imbalance between profibrotic and antifibrotic mechanisms causes the continuous activation of proliferation, contractility, and the migration of myofibroblasts, resulting in the overproduction of extracellular matrix (45, 46). High mobility group box-1 protein is a DAMP that plays a vital role in liver disease and can interact with toll-like receptor 4 (TLR4) to promote immune responses and inflammation (47–49). TLR4 can stimulate hematopoietic stem cells to produce chemokines and express adhesion molecules. It can also promote inflammatory cell infiltration into the liver, activate the NF-κB pathway of hematopoietic stem cells, and regulate histone deacetylase-1 transcription, thereby downregulating activin membrane binding inhibitor (BAMBI) and decoy receptor for transforming growth factor-β receptor (BMP). BMP enhances the transforming growth factor (TGF)-β signaling pathway in HSCs and promotes a fibrotic response (50, 51). DHZCP can block cross-linking between TLR4 and myeloid differentiation factor 88 (MYD88), blocking the TLR4/MYD88-related signaling pathway. DHZCP can upregulate BAMBI, inhibit NF-κB activation, decrease the sensitivity of HSCs to TGF-β, and indirectly block the interaction between DAMPs and TLR4 (15). TGF-β1 and fibronectin expression are downregulated, thus blocking the process of hepatic fibrosis. Some scholars believe that anticoagulation is an essential component of hepatic fibrosis treatment (51, 52). DHZCP can alleviate hepatic fibrosis by increasing GAS5 expression, suppressing p-Erk, and inhibiting p-p38 (53). DHZCP reportedly also significantly increased cardiac trophic blood flow in normal and post-vertical xerophyl-induced myocardial ischemia mice, thus widening coronary blood vessels and improving myocardial microcirculation.

It has been reported that DHZCP contains active ingredients, such as baicalin, hypoxanthine, Rhein, Rhubarbin, Rhubarbol, and Rhubarbin methyl ether. In one study, rhubarb significantly reduced ALT activity, HA, PCIII concentrations, and hepatic MDA levels in CCl4/ethanol-injured rats. Histological changes associated with hepatic fibrosis were also significantly improved in these rats, and α-SMA and TGF-β1 expression was decreased (54). Rhein improved hepatic microcirculation in rats with thioredoxin-induced hepatic fibrosis by inhibiting inducible nitric oxide synthase (iNOS) and increasing endothelial nitric oxide synthase (eNOS) expression. DHZCP improved liver microcirculation in rats with thioacetamine-induced hepatic fibrosis by inhibiting iNOS and increasing eNOS (54). Baicalin can induce NLRP3 inflammasomes and promote liver regeneration after liver injury by acetaminophen (55). Recent research indicates that DHZCP can attenuate CCl4-induced rat liver fibrosis *via* the PI3K-Akt signaling pathway, which also confirms the anti-fibrotic effect of DHZCP (56, 57). These reports suggest that DHZCP can assist the recovery of the liver microenvironment by promoting hepatic microcirculation. DHZCP has not demonstrated any significant adverse effects. It exerts significant beneficial effects on liver injury by reversing associated biochemical parameters and histopathological changes, which may be weakened with an increase in the dose (44 g kg^–1^⋅d^–1^) and a longer course of treatment (>2 months) (58–60). DHZCP is reportedly contraindicated during pregnancy (11, 12), and it requires more rigorous investigation and evaluation. The present meta-analysis demonstrates that DHZCP combined with entecavir is a beneficial clinical protocol, which supports evidence from previous studies and provides a reference with respect to medication used to treat hepatic fibrosis and its complications. The confirmed clinical protocol also enriches clinical options for the treatment of cirrhosis.

The heterogeneity apparent in the analysis of results was of serious concern and may be due to various reasons. The course of treatment, stage of disease, and treatment regimen led to an increase in heterogeneity to varying degrees. These heterogeneities may be related to the randomization methods used in the studies, allocation concealment, and ambiguity with regard to double blinding. Due to the condition of only one data being available in some of the subgroup analyses, the results need to be interpreted with caution. Based on the heterogeneity and the risk of bias, further quality control of clinical studies is needed in the future.

## Conclusion

The DHZCP-based therapy can significantly reverse hepatic fibrosis by reducing extracellular matrix, thereby improving the widening of the portal vein internal diameter and splenomegaly. It can also improve liver function by increasing the ALB and reducing the ALT levels. The therapy is appropriate for the timely treatment of hepatitis, and it is also effective for hepatic cirrhosis. DHZCP combined with entecavir is a promising clinical protocol for the treatment of hepatic fibrosis. The pharmacological mechanistic studies indicate that DHZCP can interfere with the proliferation and activation of HSCs *via* multiple pathways, alleviate inflammation, and block the accumulation of extracellular matrix. These observations demonstrate the value of the clinical application of DHZCP.

## Limitations

The designs of the studies included in the current meta-analysis were not rigorous enough. The significant heterogeneity evident in the studies may be due to sampling error, blinding bias, and allocation concealment bias. A representative funnel plot was asymmetrical, suggesting the possibility of significant publication bias and exaggerated treatment effects. Thus, the results of this analysis need to be interpreted with caution. More rigorously designed clinical and preclinical studies investigating the treatment of hepatic fibrosis with DHZCPs are required.

## Data availability statement

The original contributions presented in this study are included in the article/Supplementary material, further inquiries can be directed to the corresponding author.

## Author contributions

ZY and QH contributed to the conception and design of the study and wrote the first draft of the manuscript. YS, AW, and YH searched the database separately. MW, DM, and KQ performed the selection of studies separately. LL, CZ, and XZ carried out the statistical analysis. QY was responsible for technical guidance and quality control of the manuscript. All authors contributed to manuscript revision, read, and approved the submitted version. All divergences were resolved through discussion among all authors.

## Footnotes

1
http://www.chictr.org.cn/

2
http://www.clinicaltrials.gov/

## References

[1] Scaglione S Kliethermes S Cao G Shoham D Durazo R Luke A The epidemiology of cirrhosis in the united states: A population-based study. *J Clin Gastroenterol.* (2015) 49:690–6. 10.1097/MCG.0000000000000208 25291348

[2] Blachier M Leleu H Peck-Radosavljevic M Valla DC Roudot-Thoraval F. The burden of liver disease in Europe: A review of available epidemiological data. *J Hepatol.* (2013) 58:593–608. 10.1016/j.jhep.2012.12.005 23419824

[3] Spearman CW Sonderup MW. Health disparities in liver disease in sub-Saharan Africa. *Liver Int.* (2015) 35:2063–71. 10.1111/liv.12884 26053588

[4] Lee YA Wallace MC Friedman SL. Pathobiology of liver fibrosis: A translational success story. *Gut.* (2015) 64:830–41. 10.1136/gutjnl-2014-306842 25681399 PMC4477794

[5] Roehlen N Crouchet E Baumert TF. Liver fibrosis: Mechanistic concepts and therapeutic perspectives. *Cells-Basel.* (2020) 9:875. 10.3390/cells9040875 32260126 PMC7226751

[6] Buti M Riveiro-Barciela M Esteban R. Long-term safety and efficacy of nucleo(t)side analogue therapy in hepatitis B. *Liver Int.* (2018) 38(Suppl 1):84–9. 10.1111/liv.13641 29427500

[7] Wang XN Tao Q Feng Q Peng JH Liu P Hu YY. [Effects of chinese herbal medicine yiguanjian decoction on collagen metabolism of hepatic tissues in rats with ccl4-induced liver fibrosis]. *Zhong Xi Yi Jie He Xue Bao.* (2011) 9:651–7. 10.3736/jcim20110612 21669170

[8] Shan L Liu Z Ci L Shuai C Lv X Li J. Research progress on the anti-hepatic fibrosis action and mechanism of natural products. *Int Immunopharmacol.* (2019) 75:105765. 10.1016/j.intimp.2019.105765 31336335

[9] Tian H Liu L Li Z Liu W Sun Z Xu Y Chinese medicine CGA formula ameliorates liver fibrosis induced by carbon tetrachloride involving inhibition of hepatic apoptosis in rats. *J Ethnopharmacol.* (2019) 232:227–35. 10.1016/j.jep.2018.11.027 30471378

[10] Kong D Zhang Z Chen L Huang W Zhang F Wang L Curcumin blunts epithelial-mesenchymal transition of hepatocytes to alleviate hepatic fibrosis through regulating oxidative stress and autophagy. *Redox Biol.* (2020) 36:101600. 10.1016/j.redox.2020.101600 32526690 PMC7287144

[11] Barolet R Bensky D. *Chinese herbal medicine: formulas & strategies.* New York, NY: SYRAWOOD Publishing House (1990). 562 p.

[12] Xu E Ye Q. *Chinese Medicinal Formulas.* 3rd ed. Beijing: People’s Medical Publishing House (2021).

[13] Hou S Bin C. Postoperative effect observation and clinical study of dahuang zhechong pills from jingui yaolue in treating patients with early-to-mid prostate cancer undergoing radical resection. *Comput Intell Neurosci.* (2022) 2022:2998825. 10.1155/2022/2998825 35528360 PMC9068324

[14] Tang WY Liang JT Wu J Liu L Lu MZ He XY Efficacy and safety of dahuang zhechong pill in silicosis: a randomized controlled trial. *Evid Based Complement Alternat Med.* (2021) 2021:4354054. 10.1155/2021/4354054 34840587 PMC8616670

[15] Yuan HX. [Clinical application of Dahuang Zhechong Pill in gynecology]. *J Chin Integrative Med.* (2009) 7:168–70. 10.3736/jcim20090214 19216862

[16] Liu XD Xu XJ Zhao ZZ Lu P. Effect of Dahuang Zhechong pill on cross-linking of lipopolysaccharide with TLR4 in hepatic stellate cells. *Guangdong Med J.* (2015) 40:176–9.

[17] Son JH Lee SS Lee Y Kang BK Sung YS Jo S Assessment of liver fibrosis severity using computed tomography-based liver and spleen volumetric indices in patients with chronic liver disease. *Eur Radiol.* (2020) 30:3486–96. 10.1007/s00330-020-06665-4 32055946

[18] Guo JJ Tian ZA Li YN. Clinical effects of Dahuang Zhechong pill combined with tenofovir for the treatment of chronic hepatitis B liver fibrosis. *Chin J Postgra Med.* (2020) 43:1109–14.

[19] Pan W Li JH. Clinical study of Dahuang Zhechong pill in combination with entecavir for hepatitis B cirrhosis. *Mod Dig Intervent.* (2019) 4:1947.

[20] Page MJ McKenzie JE Bossuyt PM Boutron I Hoffmann TC Mulrow CD The PRISMA 2020 statement: An updated guideline for reporting systematic reviews. *BMJ.* (2021) 372:n71. 10.1136/bmj.n71 33782057 PMC8005924

[21] Hong HL. Clinical observation of entecavir combined with Dahuang Zhechong pill in the treatment of hepatitis B cirrhosis. *J Med Theo Prac.* (2019) 32:1003–5.

[22] Yao ZC Cai YJ Shi YM Gao DK. Effect of Dahuang Zhechong Capsule combined with entecavir on liver fibrosis and T-cell subsets in pa-tients with hepatitis B cirrhosis. *Guangdong Med J.* (2019) 40:3141–6.

[23] Huang HG. Effect of Dahuang Zhechong pill combination antiviral therapy on serum viral replication indexes and immune and inflammatory indexes in patients with chronic hepatitis B cirrhosis. *J Hainan Med Coll.* (2017) 23:1048–51.

[24] Zhang HX Liu XD Wang CY. Efficacy of Dahuang Zhechong pill in combination with entecavir in the treatment of chronic hepatitis B cirrhosis. *Chin J Integr Tradit West Med Dig.* (2016) 24:575–7.

[25] Zhou XL Wu CX Chen YH. Clinical study of Dahuang Zhechong pill in combination with entecavir for hepatitis B liver fibrosis. *Lingnan J. Emerg Med.* (2016) 21:258–9.

[26] Liu WW Yao JH Wu X Li H Tian ZA. 34 cases of liver fibrosis in chronic hepatitis B treated with Dahuang Zhechong Capsule. *China Pharm.* (2013) 24:237–8.

[27] Liu XY Liu JS. Dahuang Zhechong pill combined with western medicine for 66 cases of hepatitis B cirrhosis in compensated stage. *Henan Tradit Med J.* (2013) 33:56–7.

[28] Feng XH. Dahuang Zhechong pill combined with interferon-α for the treatment of chronic hepatitis B in 30 cases. *J Pract Tradit Chin Intern Med.* (2011) 25:51–2.

[29] Cheng MH Wu QF. Efficacy of adefovir combined with Dahuang Zhechong pill in the treatment of active hepatitis B cirrhosis. *Chin Rem Clin.* (2009) 9(Suppl 1):47–8.

[30] Liang XL. Clinical observation of Dahuang Zhechong pill in the treatment of viral hepatitis cirrhosis. *Chin Foreign Med Treat.* (2009) 28:129–30.

[31] Zhang BJ Dou Q. Efficacy of Adefovir combined with Dahuang Zhechong pill in the treatment of hepatitis B cirrhosis. *J Emerg Tradit Chin Med.* (2008) 17:611–2.

[32] Miao YD Yu JG. Dahuang Zhechong pill with lamivudine for chronic hepatitis B liver fibrosis. *J Zhejiang Chin Med Univ.* (2007) 36:695–6.

[33] Xu Q Zhu P Zhang B Du JX Li F Jiang SL. Clinical efficacy of Dahuang Zhechong pill against liver fibrosis. *Lishizhen Med Mater Med Res.* (2006) 17:808–9.

[34] Tian GQ Guo YJ. Clinical study of Dahuang Zhechong Capsule in the treatment of chronic hepatitis B. *J Jining Med Coll.* (2003) 26:74.

[35] Shen ZL Lu ZY. The efficacy of Dahuang Zhechong pill in preventing and treating liver fibrosis in chronic viral hepatitis B. *Hubei J Tradit Chin Med.* (2002) 8:21.

[36] Lin JH Liu QY Lin YS Wu C. Effect of Dahuang Zhechong pill on liver fibrosis indexes in chronic hepatitis. *Chin J Integr Tradit West Med Liver Dis.* (2001) 11:98–9.

[37] China Pharmacopoeia Committee. *Chinese pharmacopoeia.* China Pharmacopoeia Committee (2020). Beijing: Chemical industry press.

[38] Fu CK Xu HJ Zhang ZM Huang Y Chen ZP Li WD. Determination of the content of 17 quality markers in Dahuang Zhechong Pill. *China Pharm.* (2021) 32:2353–7.

[39] Li W Zi-Hui N Yun-Cong X Xi-Qiong Z Sha-Li D Ke-Xin C Investigation on the Characteristic Components of Dahuang Zhechong Pill Based on High-Performance Liquid Chromatography (HPLC) Fingerprint. *Nat Prod Commun.* (2019) 14:

[40] Wu L Du S Yang F Ni Z Chen Z Liu X Simultaneous determination of nineteen compounds of Dahuang zhechong pill in rat plasma by UHPLC-MS/MS and its application in a pharmacokinetic study. *J Chromatogr B Analyt Technol Biomed Life Sci.* (2020) 1151:122200. 10.1016/j.jchromb.2020.122200 32526664

[41] Zhang G Wang DX. Simultaneous determination of six ingredients in Dahuang Hibiscus Pill by HPLC-PDA method. *China Pharm.* (2019) 30:54–8.

[42] Tao J Peng HQ Cai WM Dong FQ Weng HL Liu RH. Influence factors of serum fibrosis markers in liver fibrosis. *World J Gastroenterol.* (2003) 9:2497–500. 10.3748/wjg.v9.i11.2497 14606083 PMC4656527

[43] Harding DJ Perera MT Chen F Olliff S Tripathi D. Portal vein thrombosis in cirrhosis: Controversies and latest developments. *World J Gastroenterol.* (2015) 21:6769–84. 10.3748/wjg.v21.i22.6769 26078553 PMC4462717

[44] Wei F Lang Y Gong D Fan Y. Effect of Dahuang zhechong formula on liver fibrosis in patients with chronichepatitis B: a meta-analysis. *Complement Ther Med.* (2015) 23:129–38. 10.1016/j.ctim.2014.12.011 25637160

[45] Ezhilarasan D Sokal E Najimi M. Hepatic fibrosis: It is time to go with hepatic stellate cell-specific therapeutic targets. *Hepatobiliary Pancreat Dis Int.* (2018) 17:192–7. 10.1016/j.hbpd.2018.04.003 29709350

[46] Zhou WC Zhang QB Qiao L. Pathogenesis of liver cirrhosis. *World J Gastroenterol.* (2014) 20:7312–24. 10.3748/wjg.v20.i23.7312 24966602 PMC4064077

[47] Mihm S. Danger-Associated molecular patterns (DAMPs): Molecular triggers for sterile inflammation in the liver. *Int J Mol Sci.* (2018) 19:3104. 10.3390/ijms19103104 30309020 PMC6213769

[48] Gardella S Andrei C Ferrera D Lotti LV Torrisi MR Bianchi ME The nuclear protein HMGB1 is secreted by monocytes via a non-classical, vesicle-mediated secretory pathway. *EMBO Rep.* (2002) 3:995–1001. 10.1093/embo-reports/kvf198 12231511 PMC1307617

[49] Tsung A Sahai R Tanaka H Nakao A Fink MP Lotze MT The nuclear factor HMGB1 mediates hepatic injury after murine liver ischemia-reperfusion. *J Exp Med.* (2005) 201:1135–43. 10.1084/jem.20042614 15795240 PMC2213120

[50] Li J Wang FP She WM Yang CQ Li L Tu CT Enhanced high-mobility group box 1 (HMGB1) modulates regulatory T cells (Treg)/T helper 17 (Th17) balance via toll-like receptor (TLR)-4-interleukin (IL)-6 pathway in patients with chronic hepatitis B. *J Viral Hepat.* (2014) 21:129–40. 10.1111/jvh.12152 24383926

[51] Seki E De Minicis S Osterreicher CH Kluwe J Osawa Y Brenner DA TLR4 enhances TGF-beta signaling and hepatic fibrosis. *Nat Med.* (2007) 13:1324–32. 10.1038/nm1663 17952090

[52] Liu C Chen X Yang L Kisseleva T Brenner DA Seki E. Transcriptional repression of the transforming growth factor beta (TGF-beta) Pseudoreceptor BMP and activin membrane-bound inhibitor (BAMBI) by Nuclear Factor kappaB (NF-kappaB) p50 enhances TGF-beta signaling in hepatic stellate cells. *J Biol Chem.* (2014) 289:7082–91. 10.1074/jbc.M113.543769 24448807 PMC3945368

[53] Davis J Caldwell SH. Healing gone wrong: Convergence of hemostatic pathways and liver fibrosis? *Clin Sci (Lond).* (2020) 134:2189–201. 10.1042/CS2019110263 32844997

[54] Guo MZ Li XS Xu HR Mei ZC Shen W Ye XF. Rhein inhibits liver fibrosis induced by carbon tetrachloride in rats. *Acta Pharmacol Sin.* (2002) 23:739–44. 12147197

[55] Gong Z Deng C Xiao H Peng Y Hu G Xiang T Effect of Dahuang Zhechong pills on long non-coding RNA growth arrest specific 5 in rat models of hepatic fibrosis. *J Tradit Chin Med.* (2018) 38:190–6. 32186058

[56] Zhao HZ. *Experimental study on the anti-liver injury effect of rhubarb acid and its mechanism.* Taiyuan: Shanxi Med Univ Master (2004) 47 p.

[57] Shi L Zhang S Huang Z Hu F Zhang T Wei M Baicalin promotes liver regeneration after acetaminophen-induced liver injury by inducing NLRP3 inflammasome activation. *Free Radic Biol Med.* (2020) 160:163–77. 10.1016/j.freeradbiomed.2020.05.012 32682928

[58] Gong Z Lin J Zheng J Wei L Liu L Peng Y Dahuang Zhechong pill attenuates CCl4-induced rat liver fibrosis via the PI3K-Akt signaling pathway. *J Cell Biochem.* (2020) 121:1431–40. 10.1002/jcb.29378 31502329

[59] Li H. Advances in anti hepatic fibrotic therapy with Traditional Chinese Medicine herbal formula. *J Ethnopharmacol.* (2020) 251:112442. 10.1016/j.jep.2019.112442 31891799

[60] Xing XY Zhao YL Jia L Kong WJ Zhong YW Wang JB Evaluation of the liver protection and toxicity of Da-Huang-Zhe-Chong pill in rats. *Pharm Biol.* (2012) 50:344–50. 10.3109/13880209.2011.604333 22103766

[61] Dingchun H Chengbiao Z Yuhai L. Effect of adefovir dipivoxil capsules combined with Dahuang Zhechong capsules in the treatment of hepatic fibrosis. *Chin Med Pharm.* (2014) 4:57–70.

