# Supplementary material for: A meritorious integrated medical regimen for hepatic fibrosis and its complications via the systematic review and meta-analysis for Dahuang Zhechong pill-based therapy
Source: Front Med (Lausanne). 2022 Oct 14;9:920062. doi: 10.3389/fmed.2022.920062 (PMC9616118; doi:10.3389/fmed.2022.920062)
Supplement: Supplementary file 1 [file Data_Sheet_1.pdf]

Supplementary Material

1 Supplementary Data

1.1 Searching words

1.1.1 CNKI:

TKA=(‘肝硬变’+’肝硬化’+’肝纤维’+’肝癌癌前病变’ +’fibrosis’ +’Cirrho*’) AND FT=‘大黄?虫’ AND TKA=('临床观察'+'clinical observation'+'疗效'+'体会'+'应用'+'评价'+'临床效果'+'临床研究'+'随机对照试验'+'randomized clinical trials'+'Randomized controlled clinical trial'+'randomized controlled trial'+'randomized controlled trials'+'randomized experiment'+'rct'+'随机对照实验'+'随机对照研究'+'随机对照') NOT TKA=('meta'+'荟萃分析'+'元分析'+'系统评价') NOT TI=('研究进展'+'指南')

1.1.2 VIP:

(M=(肝硬变+肝硬化+肝纤维+肝癌癌前病变+fibrosis+Cirrho)+R=(肝硬变+肝硬化+肝纤维+肝癌癌前病变+fibrosis+Cirrho))*U=(大黄?虫)*(M=(临床观察+clinical observation+疗效+体会+应用+评价+临床效果+临床研究+随机对照试验+randomized clinical trials+Randomized controlled clinical trial+randomized controlled trial+randomized controlled trials+randomized experiment+rct+随机对照实验+随机对照研究+随机对照)+R=(临床观察+clinical observation+疗效+体会+应用+评价+临床效果+临床研究+随机对照试验+randomized clinical trials+Randomized controlled clinical trial+randomized controlled trial+randomized controlled trials+randomized experiment+rct+随机对照实验+随机对照研究+随机对照))-M=(meta+荟萃分析+元分析+系统评价+研究进展)

1.1.3 WANFANG:

主题:("肝硬变" or "肝硬化" or "肝癌癌前病变" or "肝纤维" or "fibrosis" or "Cirrho") AND 全部:(大黄 and 虫) AND 主题:(“临床观察” or “clinical observation” or “疗效” or “体会” or “应用” or “评价” or “临床效果” or “临床研究” or “随机对照试验” or “randomized clinical trials” or “Randomized controlled clinical trial” or “randomized controlled trial” or “randomized controlled trials” or “randomized experiment” or “rct” or “随机对照实验” or “随机对照研究” or “随机对照”) NOT 主题:(“meta” or “荟萃分析” or “元分析” or “系统评价”)

1.1.4 Sinomed:

("肝硬变”[常用字段:智能] OR “肝硬化”[常用字段:智能] OR “肝癌癌前病变”[常用字段:智能] OR “肝纤维”[常用字段:智能] OR “fibrosis”[常用字段:智能] OR “Cirrho*”[常用字段:智能]) AND (“大黄?虫丸”[全部字段:智能]) NOT (“研究进展”[标题:智能])

1.1.5 Pubmed, Cochrane:

#1:(Liver[MeSH Terms]) OR (Liver[Title/Abstract]) OR (hepati?[Title/Abstract]) OR (dietary[Title/Abstract]) OR (periportal[Title/Abstract])
#2:(Cirrhosis[MeSH Terms]) OR (Cirrhosis[Title/Abstract]) OR (fibrosis[Title/Abstract]) OR (fibrous[Title/Abstract])
#3:#1 and #2

((Liver[MeSH Terms]) OR (Liver[Title/Abstract]) OR (hepati?[Title/Abstract]) OR (dietary[Title/Abstract]) OR (periportal[Title/Abstract])) AND ((Cirrhosis[MeSH Terms]) OR (Cirrhosis[Title/Abstract]) OR (fibrosis[Title/Abstract]) OR (fibrous[Title/Abstract]))
#4: (Liver Cirrhosis[MeSH Terms]) OR #3
(Liver Cirrhosis[MeSH Terms]) OR (((Liver[MeSH Terms]) OR (Liver[Title/Abstract]) OR (hepati?[Title/Abstract]) OR (dietary[Title/Abstract]) OR (periportal[Title/Abstract])) AND ((Cirrhosis[MeSH Terms]) OR (Cirrhosis[Title/Abstract]) OR (fibrosis[Title/Abstract]) OR (fibrous[Title/Abstract])))
#5:(dahuangzhechong[Title/Abstract]) OR (dahuang zhechong[Title/Abstract]) AND #4
(dahuang zhechong[Title/Abstract]) AND ((Liver Cirrhosis[MeSH Terms]) OR (((Liver[MeSH Terms]) OR (Liver[Title/Abstract]) OR (hepati?[Title/Abstract]) OR (dietary[Title/Abstract]) OR (periportal[Title/Abstract])) AND ((Cirrhosis[MeSH Terms]) OR (Cirrhosis[Title/Abstract]) OR (fibrosis[Title/Abstract]) OR (fibrous[Title/Abstract]))))

1.1.6 Embase:

#1:Search: 'liver'/exp OR 'liver*':ab,ti OR 'hepati?':ab,ti OR 'dietary':ab,ti OR 'periportal':ab,ti
#2: Search: 'fibrosis'/exp OR 'fibrosis':ab,ti OR 'Cirrhosis':ab,ti OR ' fibrous':ab,ti
#3:#1 AND #2
#4: Search: 'liver cirrhosis'/exp OR 'liver fibrosis'/exp
#5: Search:#3 OR #4
#6: Search: 'dahuang zhechong':ab,ti OR 'dahuangzhechong':ab,ti
#7:Search:#5 AND #6

1.2 The specific information of DHZCP

Name: Dahuang Zhechong Pill

Herbal medicinal product contains:

Herb	Pharmaceutical latin name	Chinese name	Processing method	Dosage
Rheum officinale Baill.	*Radix et Rhizoma Rhei*	*Dà Huáng*	Powdered	300g
Tabanus bivittatus Mats.	*Tabanus*	*Méng Chóng*	Powdered	45g
Whitmania pigra Whitman.	*Hirudo*	*Shuǐ Zhì*	Powdered	60g
Eupolyphaga sinensis Walker.	*Eupolyphaga seu Steleophaga*	*Tǔ Biē Chóng*	Powdered	30g

Holotrichia diomphalia Bates	*Holotrichia*	*Qí Cáo*	Powdered	45g
Toxicodendron vernicifluum (Stokes) F.A.Barkley	*Resina Toxicodendri*	*Gān qī*	Powdered	30g
Prunus armeniaca L.	*Semen Armeniacae Amarum*	*Xìng Rén*	Powdered	120g
Paeonia lactiflora Pal.	*Radix Paeoniae Alba; Radix Paeoniae Rubra*	*Bái Sháo*	Powdered	120g
Glycyrrhiza uralensis Fisch.	*Radix et Rhizoma Glycyrrhizae*	*Gān Căo*	Powdered	90g
Scutellaria baicalensis Georgi.	*Radix Scutellariae*	*Huáng Qín*	Powdered	60g
Prunus persica (L.) Batsch	*Semen Persicae*	*Táo Rén*	Powdered	120g
Rehjnannia glutinosa Libosch.	*Radix Rehmanniae*	*Shēng Dì Huáng*	Powdered	300g

Processing Methods: These 12 medicinals are powdered and mixed. Each 100g powder was required to add 30~45g honey. The processed powder was added water for processing and was dried to made as watered-honeyed pills. (Or each 100g powder was reqired to add 80g~100g honey. The processed powder was added water for processing and was dried to made as small honeyed pill or large honeyed pill.)

Quantity of the genuine standardised herbal preparation in the herbal medicinal product:

Herb	Dosage
Rheum officinale Baill.	300g
Tabanus bivittatus Mats.	45g
Whitmania pigra Whitman.	60g

Eupolyphaga sinensis Walker.	30g
Holotrichia diomphalia Bates	45g
Toxicodendron vernicifluum (Stokes) F.A.Barkley	30g
Prunus armeniaca L.	120g
Paeonia lactiflora Pal.	120g
Glycyrrhiza uralensis Fisch.	90g
Scutellaria baicalensis Georgi.	60g
Prunus persica (L.) Batsch	120g
Rehjnannia glutinosa Libosch.	300g

Quality control measures:

All the material considered in this study used herbal products. The production standards were in accordance with the *Chinese Pharmacopoeia*, as follows:

Total chrysophanol ($C_{15}H_{10}O_4$) and total emodin ($C_{15}H_{10}O_5$) should be more than 1.1mg per 1g in water-honeyed pill, more than 0.8mg per 1g in small honeyed pill, and more than 2.4mg per pill of large honeyed pill. The content of free chrysophanol ($C_{15}H_{10}O_4$) and free emodin ($C_{15}H_{10}O_5$) should be more than 0.7mg per 1g of water-honeyed pill, more than 0.5mg per 1g of small honeyed pill, and more than 1.6mg per pill of large honeyed pill. These chemicals are primarily found in Dà Huáng (Dired root and rhizome of *Rheum officinale* Baill.). High-performance liquid chromatography was used to make the determination (HPLC).

Chemical analyses:

The chemical analysis of Da Huang Zhe Chong Pill was reported in multiple articles. According to Li Wu et al., 20 constituents of Dahuang Zhechong Pill have been identified: hypoxanthine, allantoin, gallic acid, amygdalin, gentisic acid, paeoniflorin, verbascoside, *p*-methoxy phenylacetic acid, liquiritigenin, baicalin, naringenin, paeonol, baicalein, wogonin, aloe emodin, glycyrrhizinate, rhein, emodin, chrysophanol and physcion. Additionally, these researchers mentioned that hypoxanthine nucleoside, paeoniflorin, forsythioside A, rhodiola glucoside, formononetin, verbascoside, glycyrrhizic acid, naringenin, nutmeg acid, wogonoside, norwogonin, was identified in Da Huang Zhe Chong Pill, which produced by Beijing Tong Ren Tang Co., Ltd. Apart from the above discovery, Fu Chuankui et al. reported eight more chemicals: salidroside, hydroxy paeoniflorin,

liquiritin, isoliquiritin, cinnamic acid, apigenin, chrysin. The same findings were also noted by Zhang Guiping et al.

1.3 Selective bias of primary outcome

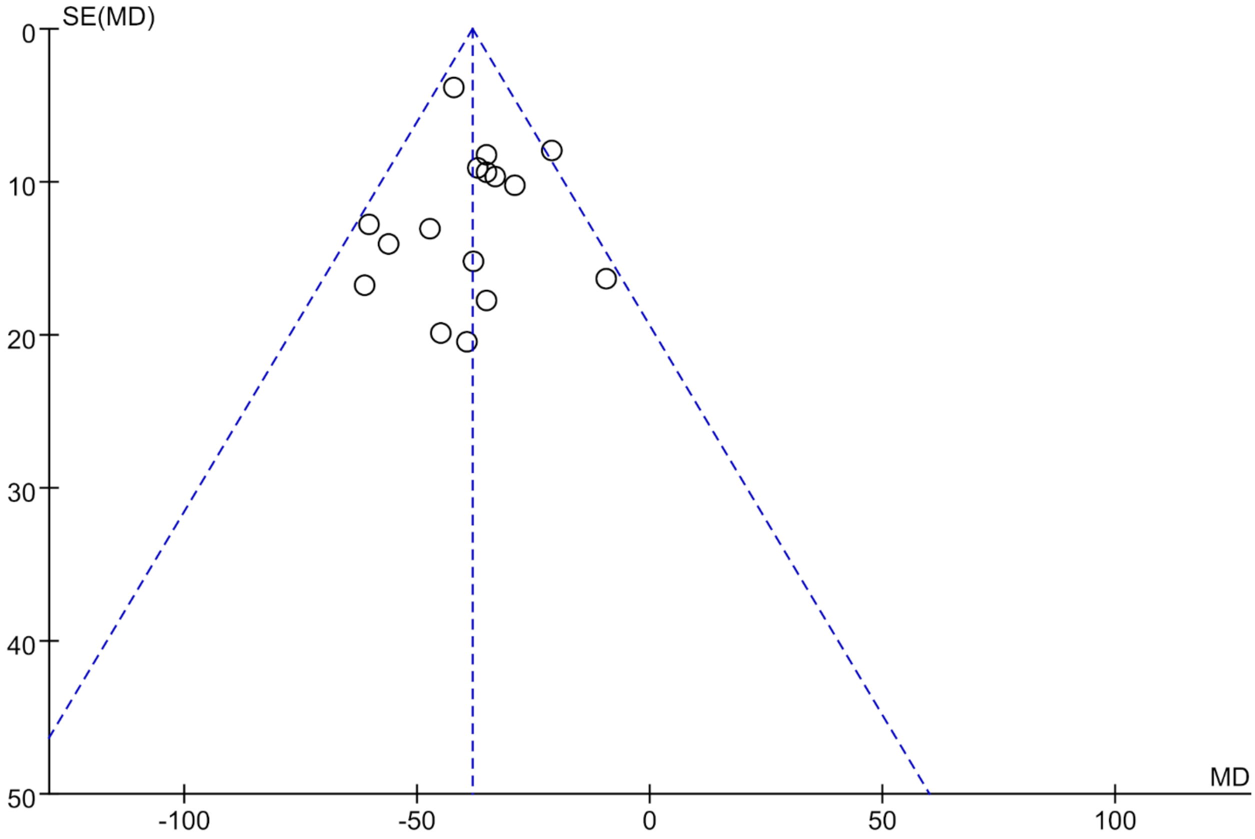

2 Supplementary Figures and Tables

2.1 Supplementary Figures

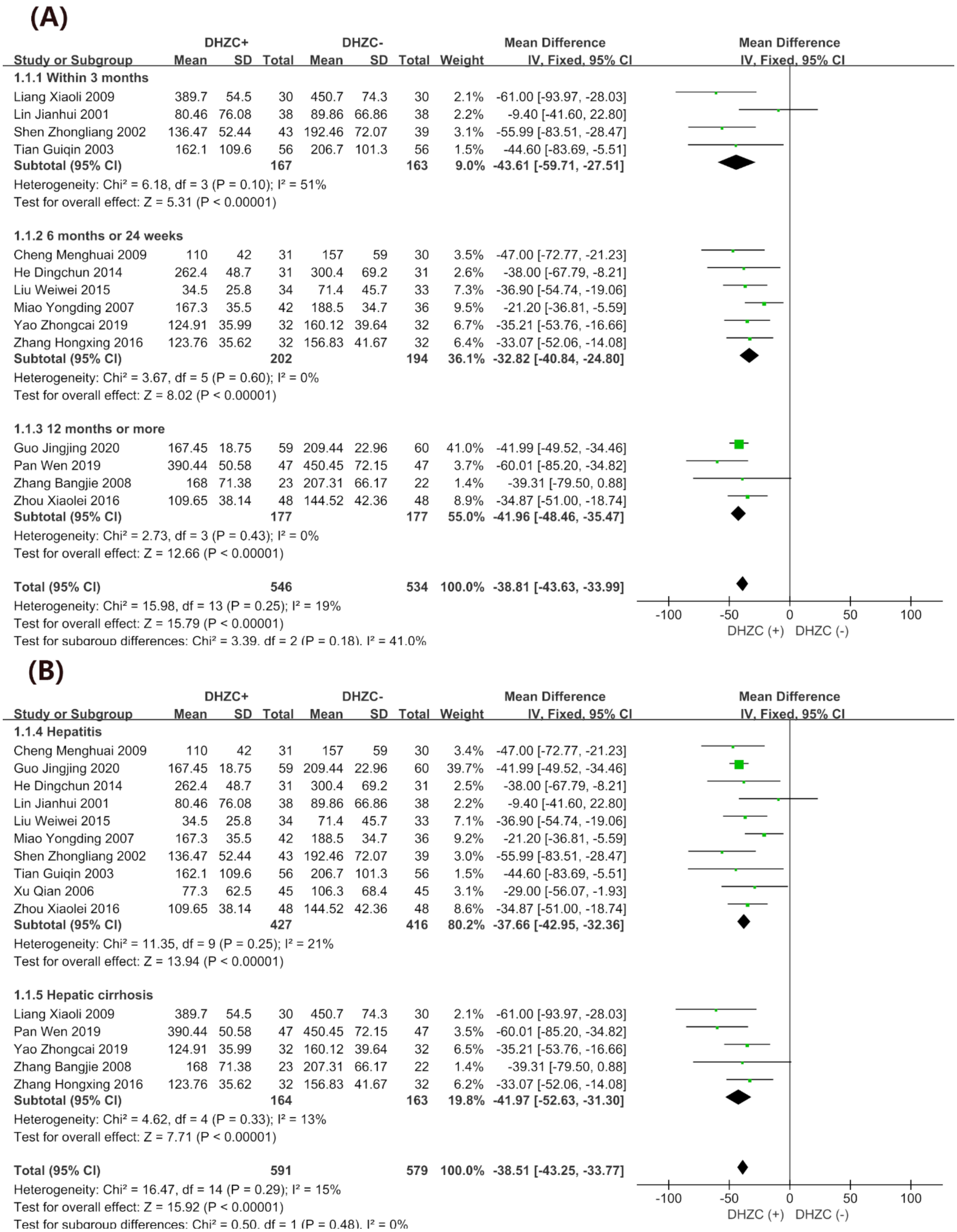

Supplementary Figure 1. The Effect of DHZCP based therapy on HA with subgroups. Fixed effects models were applied. (A) Course of treatment analysis of HA; (B) Stage of diseases analysis of HA.

(A)

Study or Subgroup	DHZC+ Mean	DHZC+ SD	DHZC+ Total	DHZC- Mean	DHZC- SD	DHZC- Total	Weight	Mean Difference IV, Fixed, 95% CI
**1.1.1 Other Therapy**								
Cheng Menghuai 2009	110	42	31	157	59	30	3.4%	-47.00 [-72.77, -21.23]
Guo Jingjing 2020	167.45	18.75	59	209.44	22.96	60	39.7%	-41.99 [-49.52, -34.46]
Liang Xiaoli 2009	389.7	54.5	30	450.7	74.3	30	2.1%	-61.00 [-93.97, -28.03]
Lin Jianhui 2001	80.46	76.08	38	89.86	66.86	38	2.2%	-9.40 [-41.60, 22.80]
Liu Weiwei 2015	34.5	25.8	34	71.4	45.7	33	7.1%	-36.90 [-54.74, -19.06]
Miao Yongding 2007	167.3	35.5	42	188.5	34.7	36	9.2%	-21.20 [-36.81, -5.59]
Pan Wen 2019	390.44	50.58	47	450.45	72.15	47	3.5%	-60.01 [-85.20, -34.82]
Shen Zhongliang 2002	136.47	52.44	43	192.46	72.07	39	3.0%	-55.99 [-83.51, -28.47]
Tian Guiqin 2003	162.1	109.6	56	206.7	101.3	56	1.5%	-44.60 [-83.69, -5.51]
Xu Qian 2006	77.3	62.5	45	106.3	68.4	45	3.1%	-29.00 [-56.07, -1.93]
**Subtotal (95% CI)**			**425**			**414**	**74.7%**	**-39.68 [-45.16, -34.19]**
Heterogeneity: Chi² = 15.66, df = 9 (P = 0.07); I² = 43%								
Test for overall effect: Z = 14.17 (P < 0.00001)								
**1.1.4 Entecavir**								
Yao Zhongcai 2019	124.91	35.99	32	160.12	39.64	32	6.5%	-35.21 [-53.76, -16.66]
Zhang Hongxing 2016	123.76	35.62	32	156.83	41.67	32	6.2%	-33.07 [-52.06, -14.08]
Zhou Xiaolei 2016	109.65	38.14	48	144.52	42.36	48	8.6%	-34.87 [-51.00, -18.74]
**Subtotal (95% CI)**			**112**			**112**	**21.4%**	**-34.45 [-44.70, -24.20]**
Heterogeneity: Chi² = 0.03, df = 2 (P = 0.99); I² = 0%								
Test for overall effect: Z = 6.59 (P < 0.00001)								
**1.1.5 Adefovir**								
He Dingchun 2014	262.4	48.7	31	300.4	69.2	31	2.5%	-38.00 [-67.79, -8.21]
Zhang Bangjie 2008	168	71.38	23	207.31	66.17	22	1.4%	-39.31 [-79.50, 0.88]
**Subtotal (95% CI)**			**54**			**53**	**3.9%**	**-38.46 [-62.40, -14.53]**
Heterogeneity: Chi² = 0.00, df = 1 (P = 0.96); I² = 0%								
Test for overall effect: Z = 3.15 (P = 0.002)								
**Total (95% CI)**			**591**			**579**	**100.0%**	**-38.51 [-43.25, -33.77]**
Heterogeneity: Chi² = 16.47, df = 14 (P = 0.29); I² = 15%								
Test for overall effect: Z = 15.92 (P < 0.00001)								
Test for subgroup differences: Chi² = 0.78, df = 2 (P = 0.68), I² = 0%								

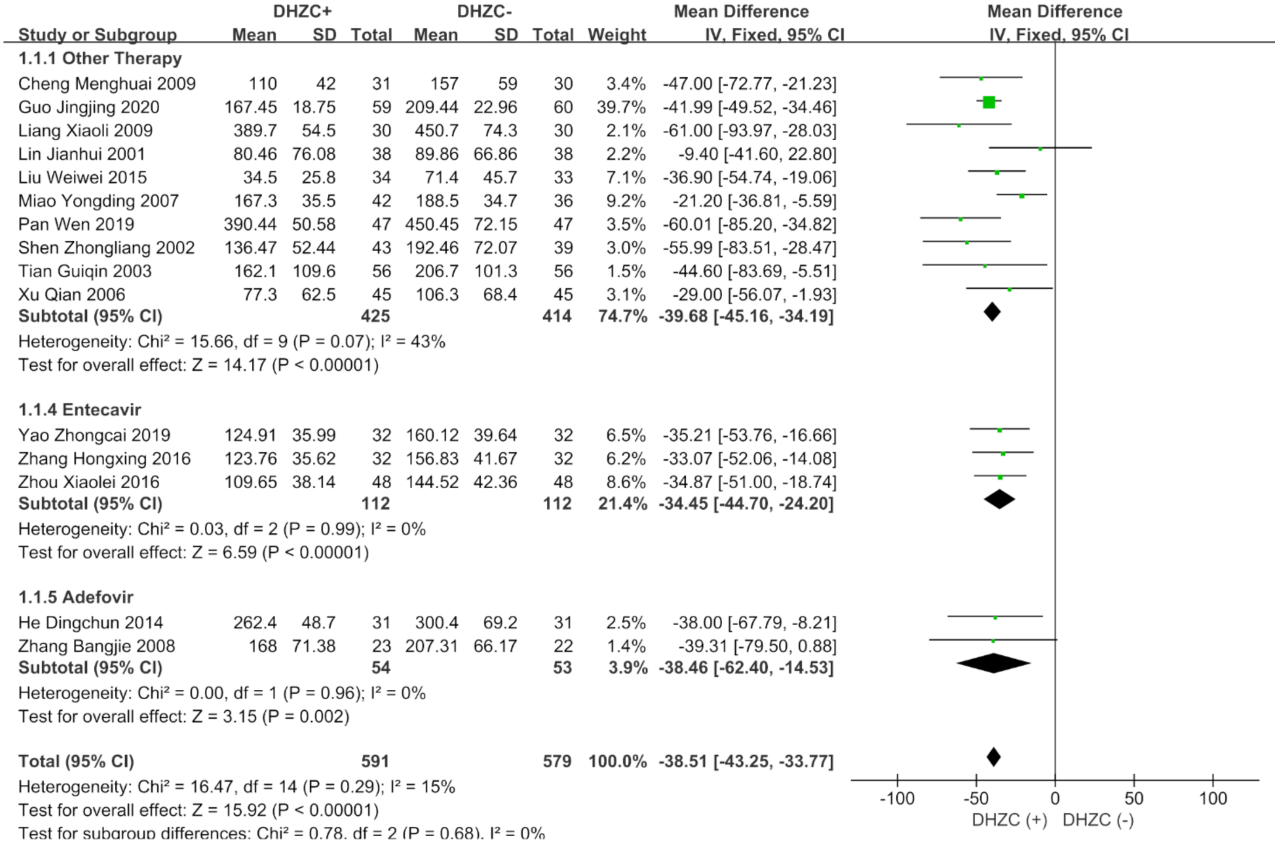

(B)

Study or Subgroup	DHZC+ Mean	DHZC+ SD	DHZC+ Total	DHZC- Mean	DHZC- SD	DHZC- Total	Weight	Mean Difference IV, Random, 95% CI
**1.2.1 Within 3 months**								
Liang Xiaoli 2009	103.8	13.9	30	118.3	21.7	30	8.6%	-14.50 [-23.72, -5.28]
Lin Jianhui 2001	139.93	48.8	38	138.64	67.86	38	7.1%	1.29 [-25.29, 27.87]
Shen Zhongliang 2002	94.08	40.1	43	98.99	12.14	39	8.4%	-4.91 [-17.49, 7.67]
Tian Guiqin 2003	153.2	157.1	56	186	13.27	56	5.6%	-32.80 [-74.09, 8.49]
**Subtotal (95% CI)**			**167**			**163**	**29.6%**	**-10.61 [-18.56, -2.66]**
Heterogeneity: Tau² = 8.11; Chi² = 3.35, df = 3 (P = 0.34); I² = 10%								
Test for overall effect: Z = 2.62 (P = 0.009)								
**1.2.2 6 months or 24 weeks**								
Cheng Menghuai 2009	102	31	31	121	40	30	8.0%	-19.00 [-37.00, -1.00]
He Dingchun 2014	181.4	48.5	31	291.3	61.6	31	7.0%	-109.90 [-137.50, -82.30]
Liu Weiwei 2015	67.5	55.7	34	98.9	71.5	33	6.7%	-31.40 [-62.15, -0.65]
Miao Yongding 2007	87.2	35.4	42	147.2	27.7	36	8.3%	-60.00 [-74.02, -45.98]
Yao Zhongcai 2019	145.78	38.04	32	181.35	47.65	32	7.7%	-35.57 [-56.70, -14.44]
Zhang Hongxing 2016	92.16	26.32	32	116.75	30.22	32	8.3%	-24.59 [-38.47, -10.71]
**Subtotal (95% CI)**			**202**			**194**	**45.8%**	**-45.81 [-68.66, -22.95]**
Heterogeneity: Tau² = 697.62; Chi² = 42.70, df = 5 (P < 0.00001); I² = 88%								
Test for overall effect: Z = 3.93 (P < 0.0001)								
**1.2.3 12 months or more**								
Guo Jingjing 2020	108.41	12.72	59	169.41	19.22	60	8.7%	-61.00 [-66.85, -55.15]
Zhang Bangjie 2008	109.39	30.47	23	112.45	28.19	22	8.0%	-3.06 [-20.20, 14.08]
Zhou Xiaolei 2016	141.26	49.58	48	181.36	51.35	48	7.7%	-40.10 [-60.29, -19.91]
**Subtotal (95% CI)**			**130**			**130**	**24.5%**	**-35.25 [-72.11, 1.60]**
Heterogeneity: Tau² = 998.44; Chi² = 41.25, df = 2 (P < 0.00001); I² = 95%								
Test for overall effect: Z = 1.87 (P = 0.06)								
**Total (95% CI)**			**499**			**487**	**100.0%**	**-33.10 [-49.07, -17.13]**
Heterogeneity: Tau² = 750.87; Chi² = 178.73, df = 12 (P < 0.00001); I² = 93%								
Test for overall effect: Z = 4.06 (P < 0.0001)								
Test for subgroup differences: Chi² = 9.31, df = 2 (P = 0.010), I² = 78.5%								

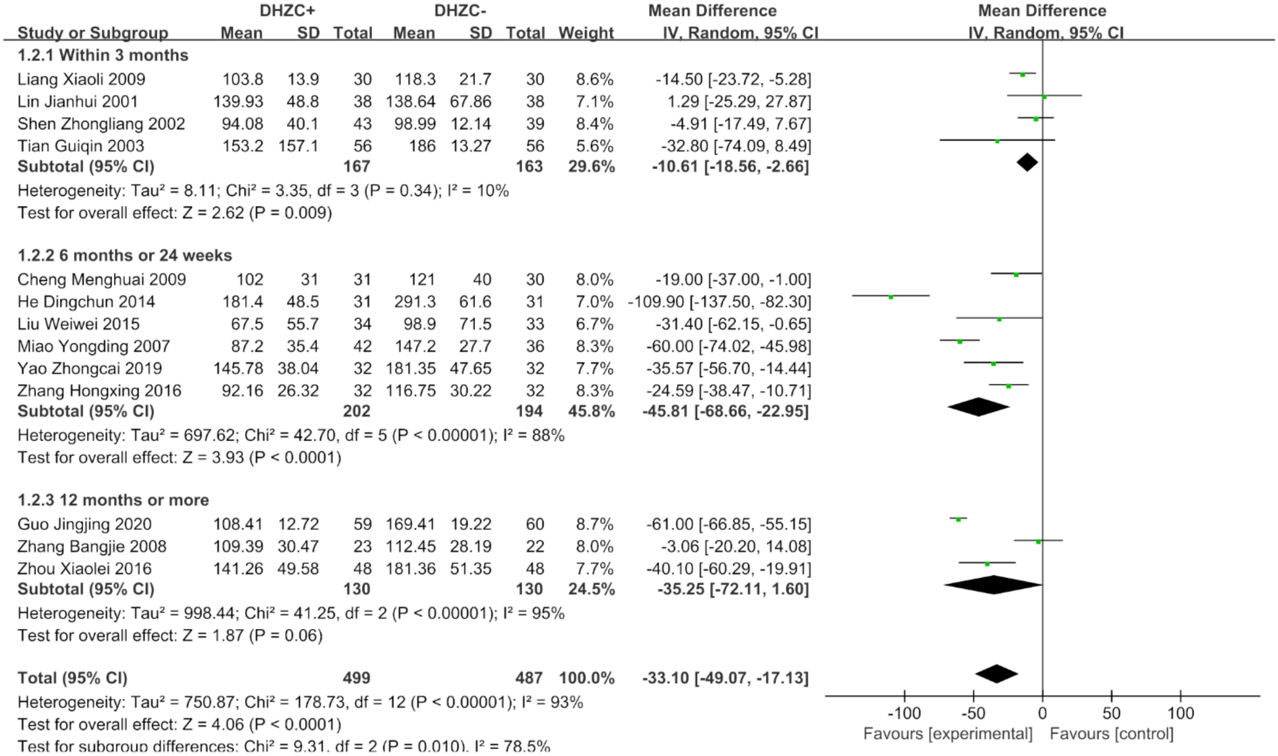

Supplementary Figure 2. The Effect of DHZCP based therapy on HA and LN. (A) HA with a subgroup of medication use for adefovir, adefovir and other therapies. Fixed effects models were applied; (B) LN with a subgroup of course of treatment for 3-, 6-, and 12 months. Random effects models were applied.

(A)

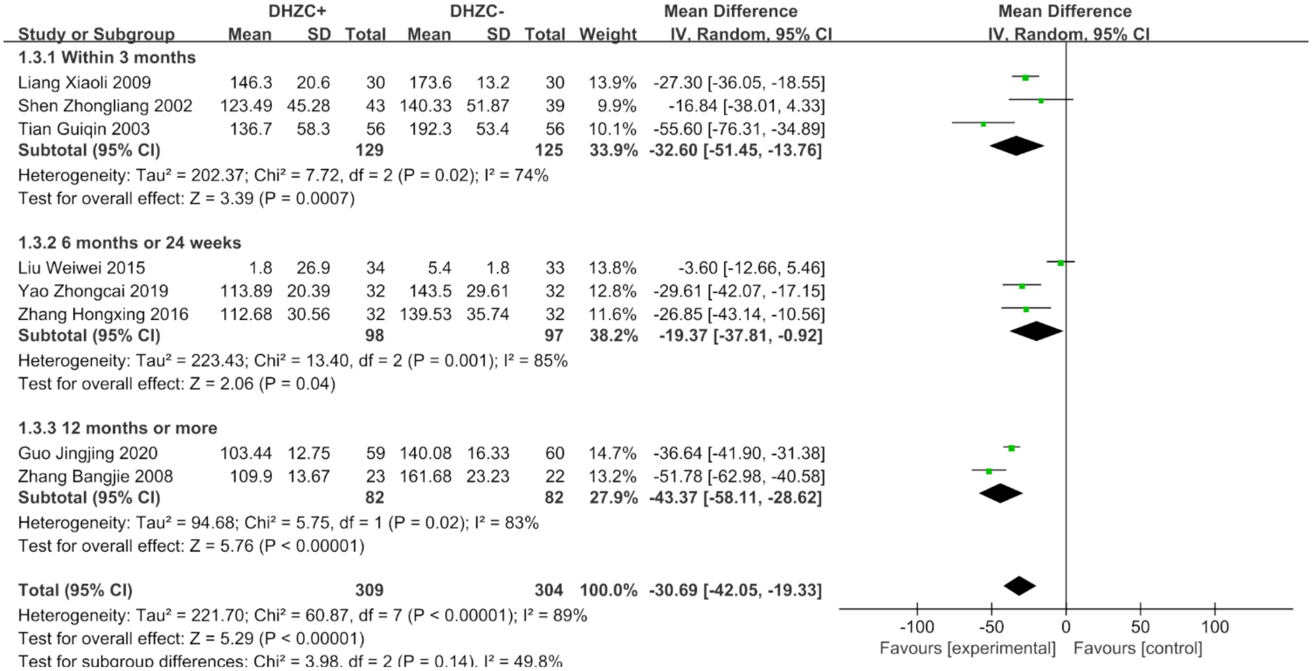

(B)

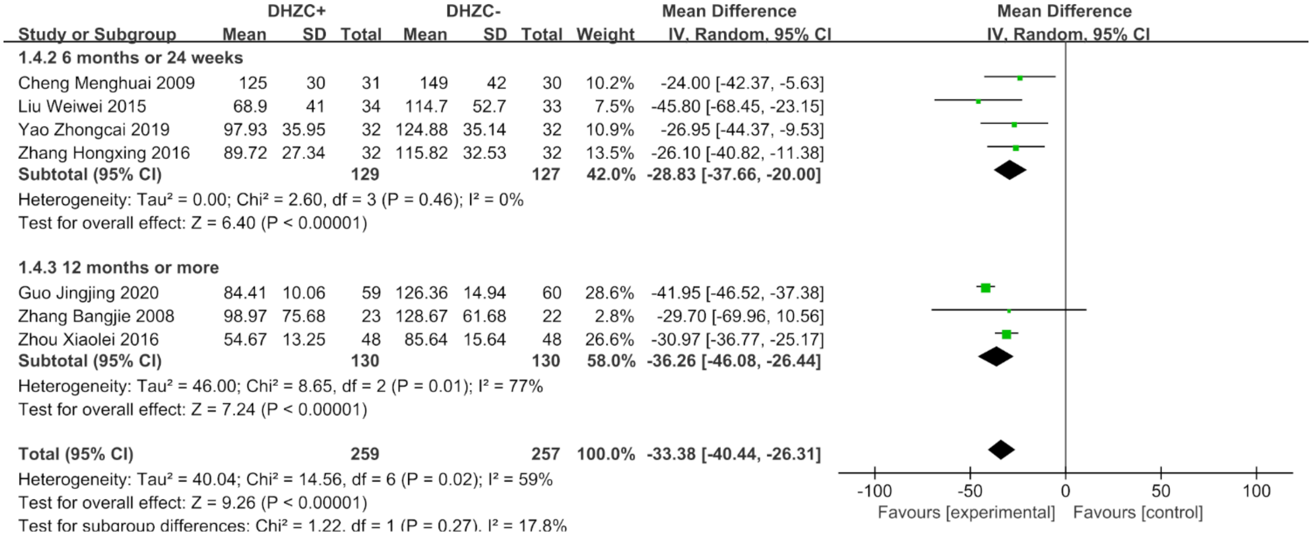

Supplementary Figure 3. The Effect of DHZCP based therapy on PCIII and ⅣC with a subgroup of course of treatment for 3-, 6- and 12-month. (A) PCIII with a subgroup of course of treatment; (B) ⅣC with a subgroup of course of treatment.

(A)

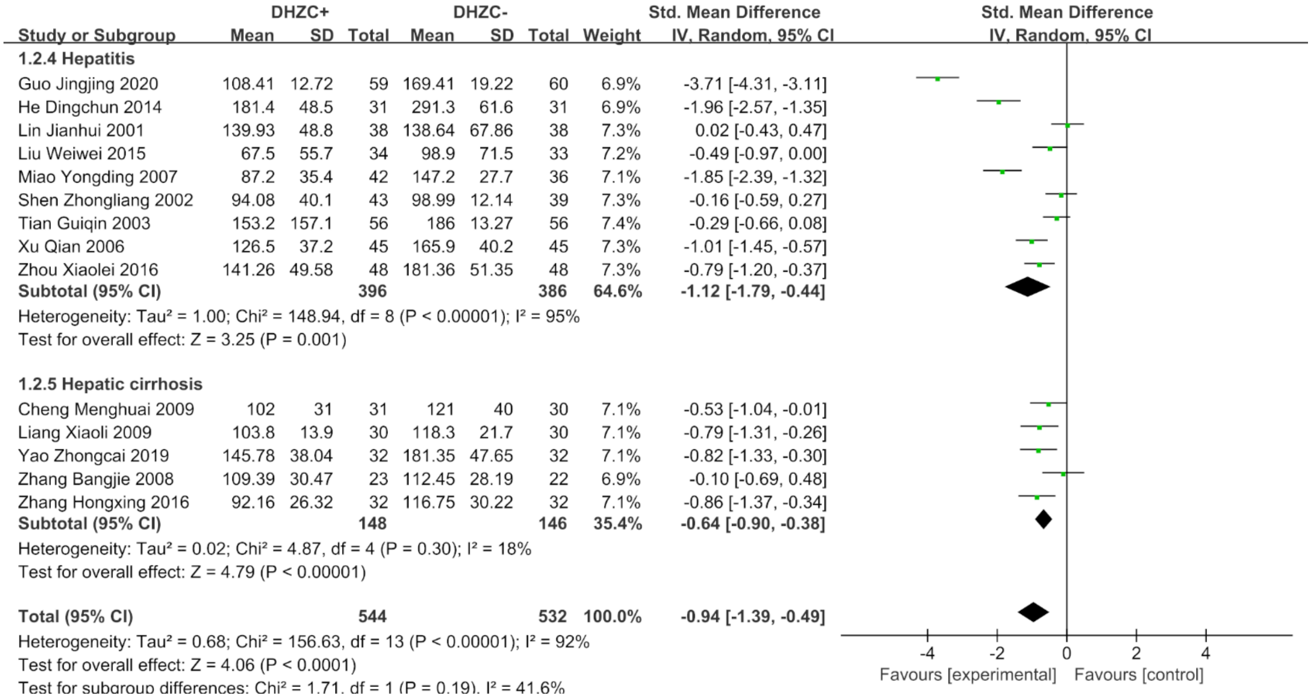

(B)

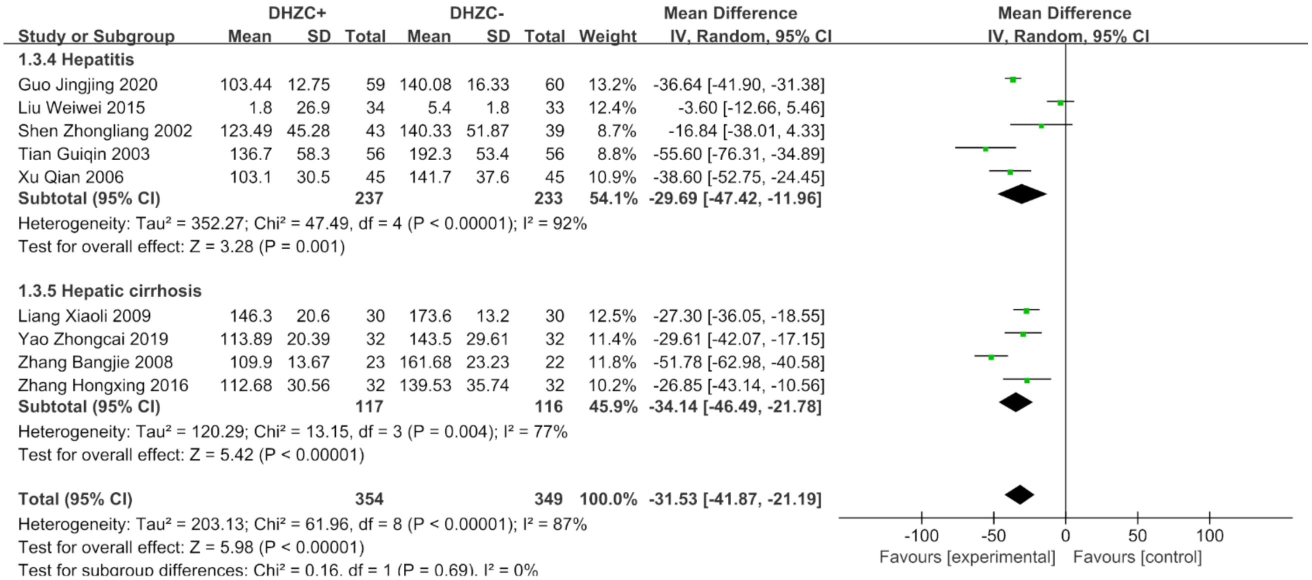

Supplementary Figure 4. The Effect of DHZCP based therapy on LN and PCⅢ with a subgroup of stage of disease for hepatitis and hepatic cirrhosis. Random effects models were applied. (A) LN; (B) PCⅢ.

(A)

Study or Subgroup	DHZC+ Mean	DHZC+ SD	DHZC+ Total	DHZC- Mean	DHZC- SD	DHZC- Total	Weight	Mean Difference IV, Random, 95% CI
**1.4.4 Hepatitis**								
Guo Jingjing 2020	84.41	10.06	59	126.36	14.94	60	20.5%	-41.95 [-46.52, -37.38]
Lin Jianhui 2001	73.71	32.83	38	81.36	54.02	38	7.5%	-7.65 [-27.75, 12.45]
Liu Weiwei 2015	68.9	41	34	114.7	52.7	33	6.3%	-45.80 [-68.45, -23.15]
Xu Qian 2006	57.8	19.1	45	91.7	27.3	45	15.4%	-33.90 [-43.63, -24.17]
Zhou Xiaolei 2016	54.67	13.25	48	85.64	15.64	48	19.4%	-30.97 [-36.77, -25.17]
**Subtotal (95% CI)**			**224**			**224**	**69.2%**	**-33.53 [-42.21, -24.85]**
Heterogeneity: Tau² = 62.97; Chi² = 17.78, df = 4 (P = 0.001); I² = 78%								
Test for overall effect: Z = 7.57 (P < 0.00001)								
**1.4.5 Hepatic cirrhosis**								
Cheng Menghuai 2009	125	30	31	149	42	30	8.4%	-24.00 [-42.37, -5.63]
Yao Zhongcai 2019	97.93	35.95	32	124.88	35.14	32	9.0%	-26.95 [-44.37, -9.53]
Zhang Bangjie 2008	98.97	75.68	23	128.67	61.68	22	2.5%	-29.70 [-69.96, 10.56]
Zhang Hongxing 2016	89.72	27.34	32	115.82	32.53	32	10.9%	-26.10 [-40.82, -11.38]
**Subtotal (95% CI)**			**118**			**116**	**30.8%**	**-26.00 [-35.32, -16.67]**
Heterogeneity: Tau² = 0.00; Chi² = 0.09, df = 3 (P = 0.99); I² = 0%								
Test for overall effect: Z = 5.46 (P < 0.00001)								
**Total (95% CI)**			**342**			**340**	**100.0%**	**-31.36 [-38.09, -24.63]**
Heterogeneity: Tau² = 51.87; Chi² = 22.35, df = 8 (P = 0.004); I² = 64%								
Test for overall effect: Z = 9.14 (P < 0.00001)								
Test for subgroup differences: Chi² = 1.34, df = 1 (P = 0.25), I² = 25.6%								

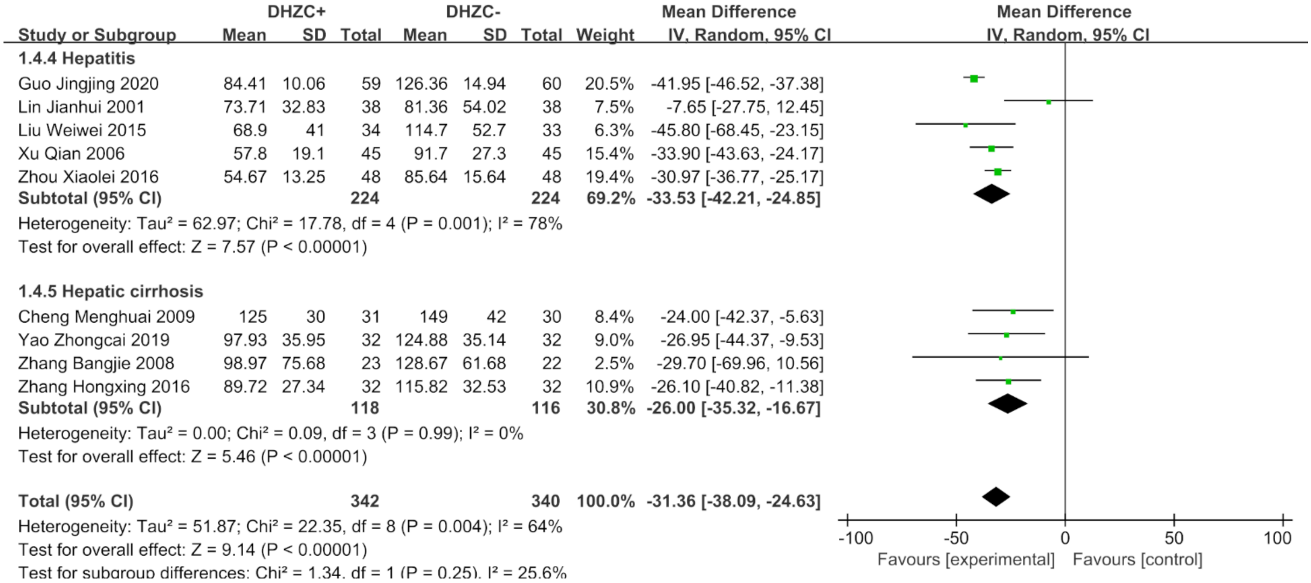

(B)

Study or Subgroup	DHZC+ Mean	DHZC+ SD	DHZC+ Total	DHZC- Mean	DHZC- SD	DHZC- Total	Weight	Mean Difference IV, Random, 95% CI
**1.2.1 Other Therapy**								
Cheng Menghuai 2009	102	31	31	121	40	30	7.4%	-19.00 [-37.00, -1.00]
Guo Jingjing 2020	108.41	12.72	59	169.41	19.22	60	8.2%	-61.00 [-66.85, -55.15]
Liang Xiaoli 2009	103.8	13.9	30	118.3	21.7	30	8.0%	-14.50 [-23.72, -5.28]
Lin Jianhui 2001	139.93	48.8	38	138.64	67.86	38	6.5%	1.29 [-25.29, 27.87]
Liu Weiwei 2015	67.5	55.7	34	98.9	71.5	33	6.1%	-31.40 [-62.15, -0.65]
Miao Yongding 2007	87.2	35.4	42	147.2	27.7	36	7.7%	-60.00 [-74.02, -45.98]
Shen Zhongliang 2002	94.08	40.1	43	98.99	12.14	39	7.8%	-4.91 [-17.49, 7.67]
Tian Guiqin 2003	153.2	157.1	56	186	13.27	56	5.0%	-32.80 [-74.09, 8.49]
Xu Qian 2006	126.5	37.2	45	165.9	40.2	45	7.5%	-39.40 [-55.40, -23.40]
**Subtotal (95% CI)**			**378**			**367**	**64.2%**	**-29.55 [-48.29, -10.81]**
Heterogeneity: Tau² = 712.87; Chi² = 131.39, df = 8 (P < 0.00001); I² = 94%								
Test for overall effect: Z = 3.09 (P = 0.002)								
**1.2.4 Entecavir**								
Yao Zhongcai 2019	145.78	38.04	32	181.35	47.65	32	7.1%	-35.57 [-56.70, -14.44]
Zhang Hongxing 2016	92.16	26.32	32	116.75	30.22	32	7.7%	-24.59 [-38.47, -10.71]
Zhou Xiaolei 2016	141.26	49.58	48	181.36	51.35	48	7.2%	-40.10 [-60.29, -19.91]
**Subtotal (95% CI)**			**112**			**112**	**21.9%**	**-30.93 [-40.99, -20.87]**
Heterogeneity: Tau² = 0.00; Chi² = 1.78, df = 2 (P = 0.41); I² = 0%								
Test for overall effect: Z = 6.03 (P < 0.00001)								
**1.2.5 Adefovir**								
He Dingchun 2014	181.4	48.5	31	291.3	61.6	31	6.4%	-109.90 [-137.50, -82.30]
Zhang Bangjie 2008	109.39	30.47	23	112.45	28.19	22	7.4%	-3.06 [-20.20, 14.08]
**Subtotal (95% CI)**			**54**			**53**	**13.9%**	**-55.91 [-160.61, 48.79]**
Heterogeneity: Tau² = 5570.01; Chi² = 41.54, df = 1 (P < 0.00001); I² = 98%								
Test for overall effect: Z = 1.05 (P = 0.30)								
**Total (95% CI)**			**544**			**532**	**100.0%**	**-33.55 [-48.37, -18.72]**
Heterogeneity: Tau² = 692.63; Chi² = 178.73, df = 13 (P < 0.00001); I² = 93%								
Test for overall effect: Z = 4.44 (P < 0.00001)								
Test for subgroup differences: Chi² = 0.24, df = 2 (P = 0.89), I² = 0%								

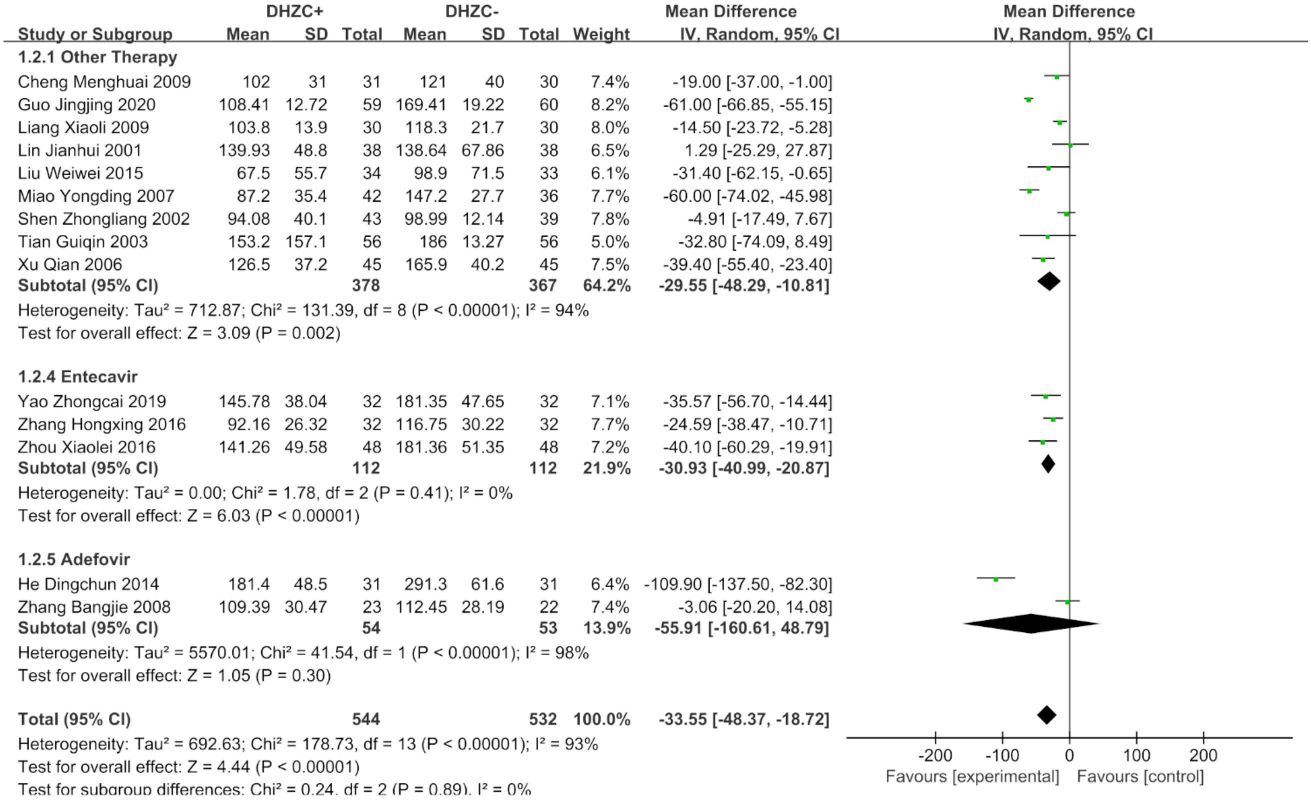

Supplementary Figure 5. The Effect of DHZCP based therapy on IVC with a subgroup of stages of disease, and LN with a subgroup of medication use. Random effects models were applied. (A) IVC with a subgroup of stages of disease; (B) LN with a subgroup of medication use.

(A)

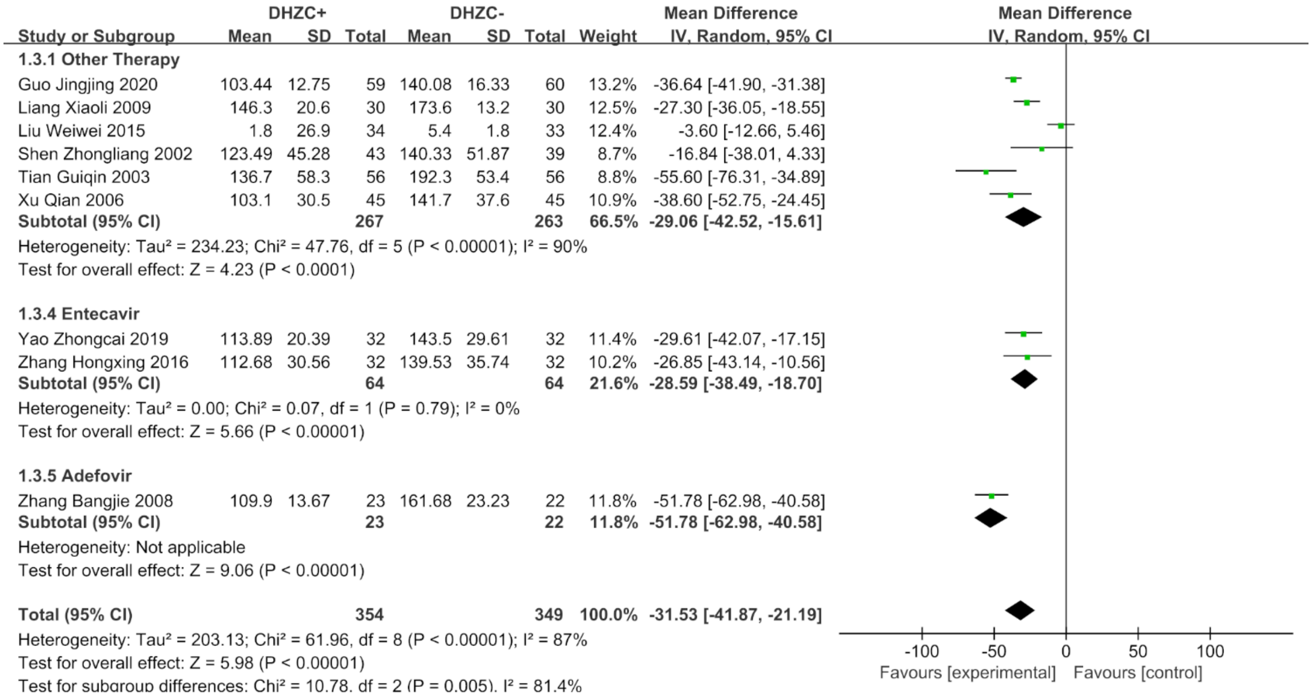

(B)

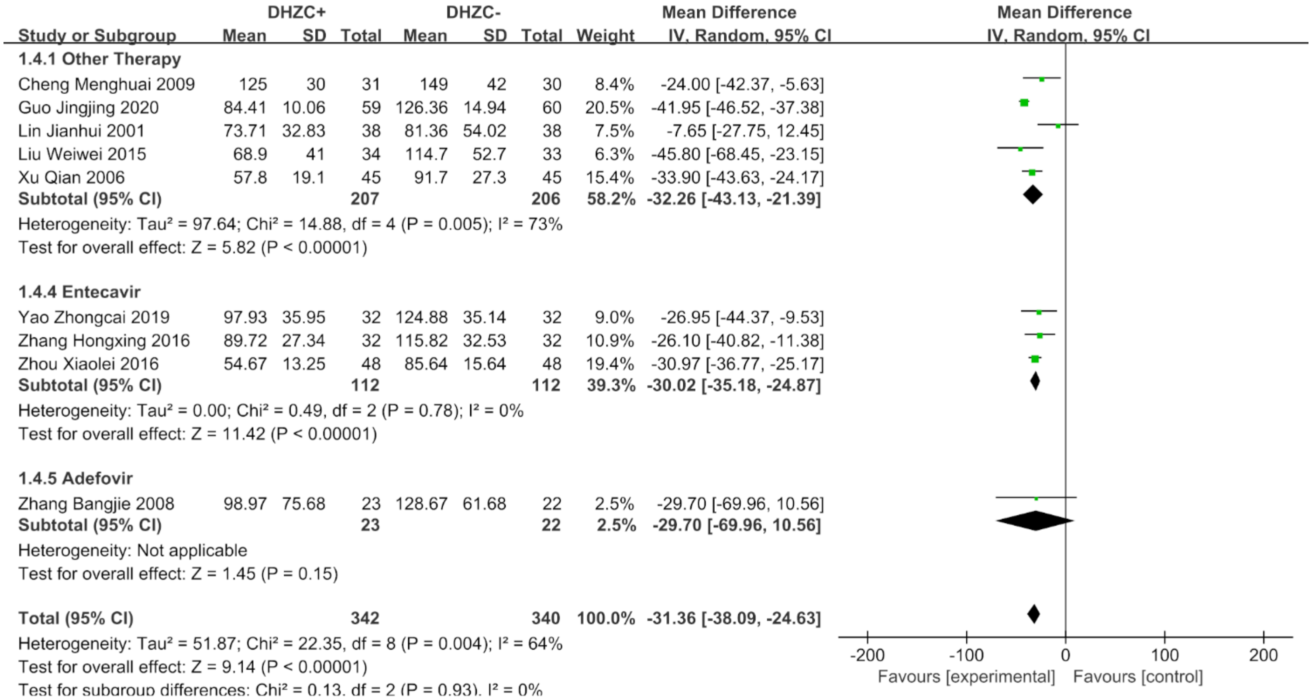

Supplementary Figure 6. The Effect of DHZCP based therapy on PCIII and IVC with a subgroup of medication use. Random effects models were applied. (A) PCIII with a subgroup of medication use; (B) IVC with a subgroup of medication use.

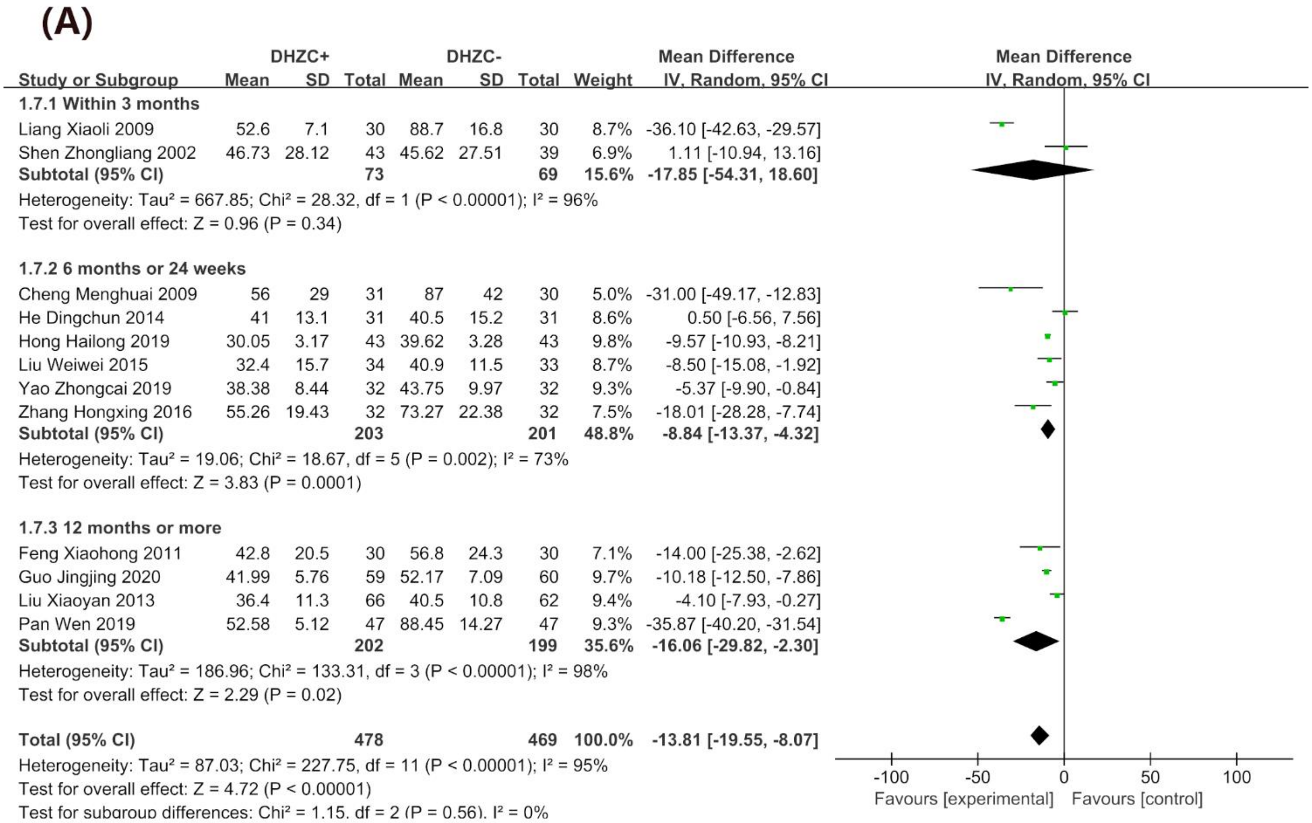

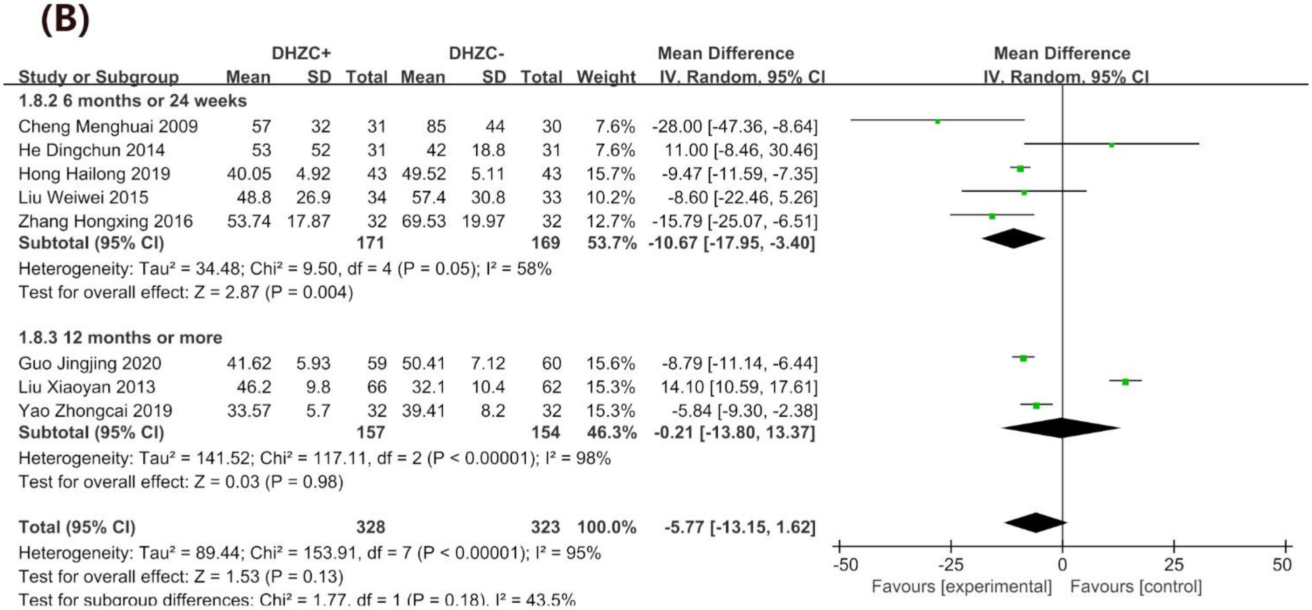

Supplementary Figure 7. The Effect of DHZCP based therapy on ALT and AST with a subgroup of course of treatment. Random effects models were applied. (A) ALT with a subgroup of course of treatment; (B) AST with a subgroup of course of treatment.

(A)

Study or Subgroup	DHZC+ Mean	SD	Total	DHZC- Mean	SD	Total	Weight	Mean Difference IV, Random, 95% CI
**1.6.2 6 months or 24 weeks**								
Cheng Menghuai 2009	38	5	31	35	3	30	19.2%	3.00 [0.94, 5.06]
He Dingchun 2014	29.2	3	31	28.6	2.9	31	25.7%	0.60 [-0.87, 2.07]
Liu Weiwei 2015	31.9	3	34	29.7	2.7	33	26.9%	2.20 [0.83, 3.57]
**Subtotal (95% CI)**			**96**			**94**	**71.8%**	**1.82 [0.49, 3.16]**
Heterogeneity: Tau² = 0.73; Chi² = 4.18, df = 2 (P = 0.12); I² = 52%								
Test for overall effect: Z = 2.67 (P = 0.007)								
**1.6.3 12 months or more**								
Liu Xiaoyan 2013	33.8	5.2	66	27.7	15.2	62	8.0%	6.10 [2.11, 10.09]
Pan Wen 2019	36.04	4.85	47	34.45	4.82	47	20.2%	1.59 [-0.36, 3.54]
**Subtotal (95% CI)**			**113**			**109**	**28.2%**	**3.50 [-0.87, 7.86]**
Heterogeneity: Tau² = 7.60; Chi² = 3.96, df = 1 (P = 0.05); I² = 75%								
Test for overall effect: Z = 1.57 (P = 0.12)								
**Total (95% CI)**			**209**			**203**	**100.0%**	**2.13 [0.87, 3.39]**
Heterogeneity: Tau² = 1.05; Chi² = 8.65, df = 4 (P = 0.07); I² = 54%								
Test for overall effect: Z = 3.31 (P = 0.0009)								
Test for subgroup differences: Chi² = 0.51, df = 1 (P = 0.47), I² = 0%								

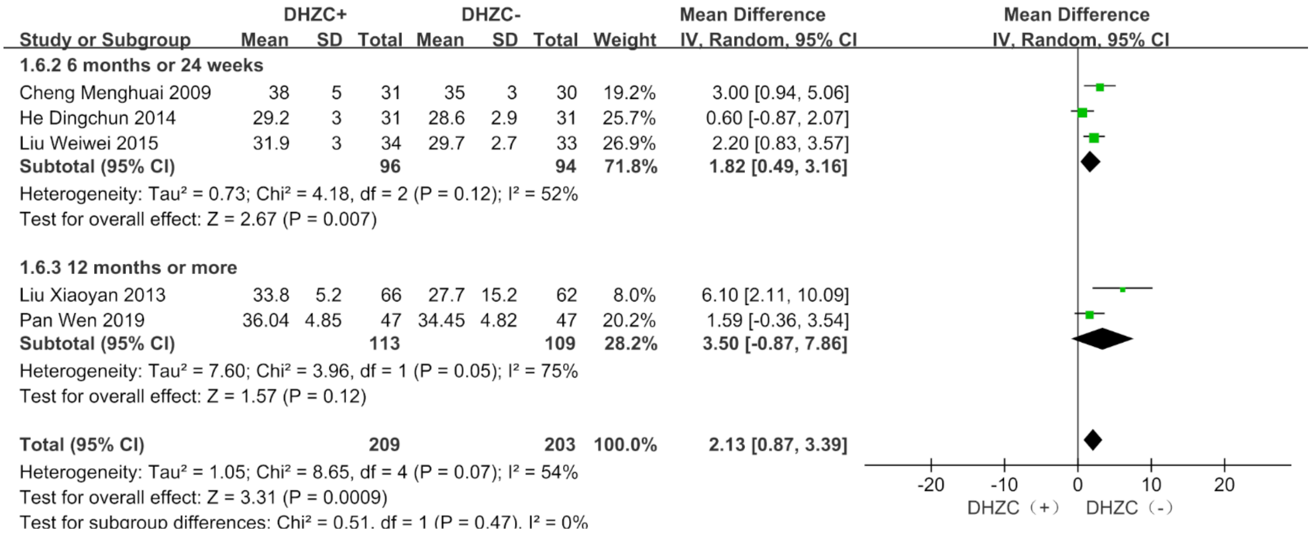

(B)

Study or Subgroup	DHZC+ Mean	SD	Total	DHZC- Mean	SD	Total	Weight	Mean Difference IV, Random, 95% CI
**1.5.2 6 months or 24 weeks**								
Cheng Menghuai 2009	28	13	31	41	14	30	10.5%	-13.00 [-19.79, -6.21]
He Dingchun 2014	65.4	23.2	31	72.5	19.8	31	6.5%	-7.10 [-17.84, 3.64]
Liu Weiwei 2015	24.5	6.8	34	21.4	5.7	33	15.6%	3.10 [0.10, 6.10]
Yao Zhongcai 2019	18.25	2.98	32	21.09	3.45	32	17.1%	-2.84 [-4.42, -1.26]
Zhang Hongxing 2016	30.52	10.63	32	39.59	12.67	32	11.9%	-9.07 [-14.80, -3.34]
**Subtotal (95% CI)**			**160**			**158**	**61.5%**	**-4.94 [-9.89, 0.02]**
Heterogeneity: Tau² = 23.80; Chi² = 28.51, df = 4 (P < 0.00001); I² = 86%								
Test for overall effect: Z = 1.95 (P = 0.05)								
**1.5.3 12 months or more**								
Guo Jingjing 2020	20.35	3.04	59	25.6	3.95	60	17.3%	-5.25 [-6.52, -3.98]
Liu Xiaoyan 2013	50.6	10.8	66	45.4	8.7	62	15.1%	5.20 [1.81, 8.59]
Pan Wen 2019	50.01	32.04	47	52.46	22.46	47	6.1%	-2.45 [-13.64, 8.74]
**Subtotal (95% CI)**			**172**			**169**	**38.5%**	**-0.70 [-9.16, 7.76]**
Heterogeneity: Tau² = 46.98; Chi² = 32.14, df = 2 (P < 0.00001); I² = 94%								
Test for overall effect: Z = 0.16 (P = 0.87)								
**Total (95% CI)**			**332**			**327**	**100.0%**	**-3.17 [-6.60, 0.26]**
Heterogeneity: Tau² = 17.26; Chi² = 63.35, df = 7 (P < 0.00001); I² = 89%								
Test for overall effect: Z = 1.81 (P = 0.07)								
Test for subgroup differences: Chi² = 0.72, df = 1 (P = 0.40), I² = 0%								

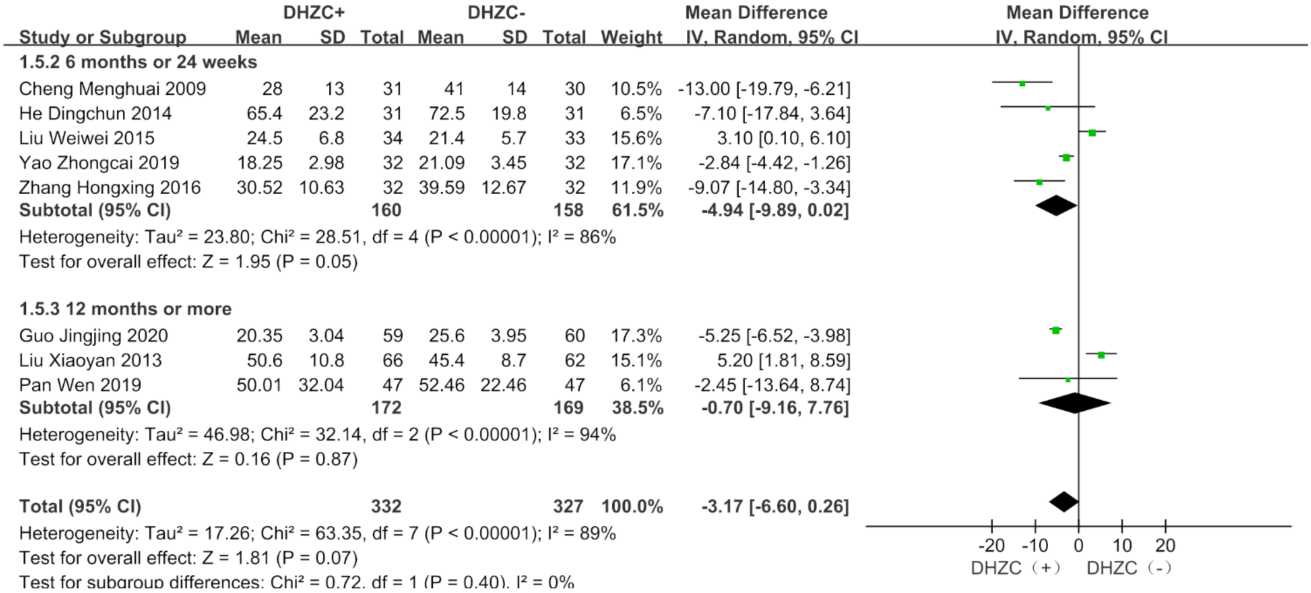

Supplementary Figure 8. The Effect of DHZCP based therapy on ALB and Tbil with a subgroup of course of treatment. Random effects models were applied. (A) ALB with a subgroup of course of treatment; (B) Tbil with a subgroup of course of treatment.

(A)

Study or Subgroup	DHZC+ Mean	DHZC+ SD	DHZC+ Total	DHZC- Mean	DHZC- SD	DHZC- Total	Weight	Mean Difference IV, Random, 95% CI
**1.8.4 Hepatitis**								
Guo Jingjing 2020	41.62	5.93	59	50.41	7.12	60	15.6%	-8.79 [-11.14, -6.44]
He Dingchun 2014	53	52	31	42	18.8	31	7.6%	11.00 [-8.46, 30.46]
Liu Weiwei 2015	48.8	26.9	34	57.4	30.8	33	10.2%	-8.60 [-22.46, 5.26]
**Subtotal (95% CI)**			**124**			**124**	**33.4%**	**-5.66 [-14.53, 3.21]**
Heterogeneity: Tau² = 32.83; Chi² = 3.91, df = 2 (P = 0.14); I² = 49%								
Test for overall effect: Z = 1.25 (P = 0.21)								
**1.8.5 Hepatic cirrhosis**								
Cheng Menghuai 2009	57	32	31	85	44	30	7.6%	-28.00 [-47.36, -8.64]
Hong Hailong 2019	40.05	4.92	43	49.52	5.11	43	15.7%	-9.47 [-11.59, -7.35]
Liu Xiaoyan 2013	46.2	9.8	66	32.1	10.4	62	15.3%	14.10 [10.59, 17.61]
Yao Zhongcai 2019	33.57	5.7	32	39.41	8.2	32	15.3%	-5.84 [-9.30, -2.38]
Zhang Hongxing 2016	53.74	17.87	32	69.53	19.97	32	12.7%	-15.79 [-25.07, -6.51]
**Subtotal (95% CI)**			**204**			**199**	**66.6%**	**-7.20 [-18.47, 4.07]**
Heterogeneity: Tau² = 146.11; Chi² = 141.05, df = 4 (P < 0.00001); I² = 97%								
Test for overall effect: Z = 1.25 (P = 0.21)								
**Total (95% CI)**			**328**			**323**	**100.0%**	**-5.77 [-13.15, 1.62]**
Heterogeneity: Tau² = 89.44; Chi² = 153.91, df = 7 (P < 0.00001); I² = 95%								
Test for overall effect: Z = 1.53 (P = 0.13)								
Test for subgroup differences: Chi² = 0.04, df = 1 (P = 0.83), I² = 0%								

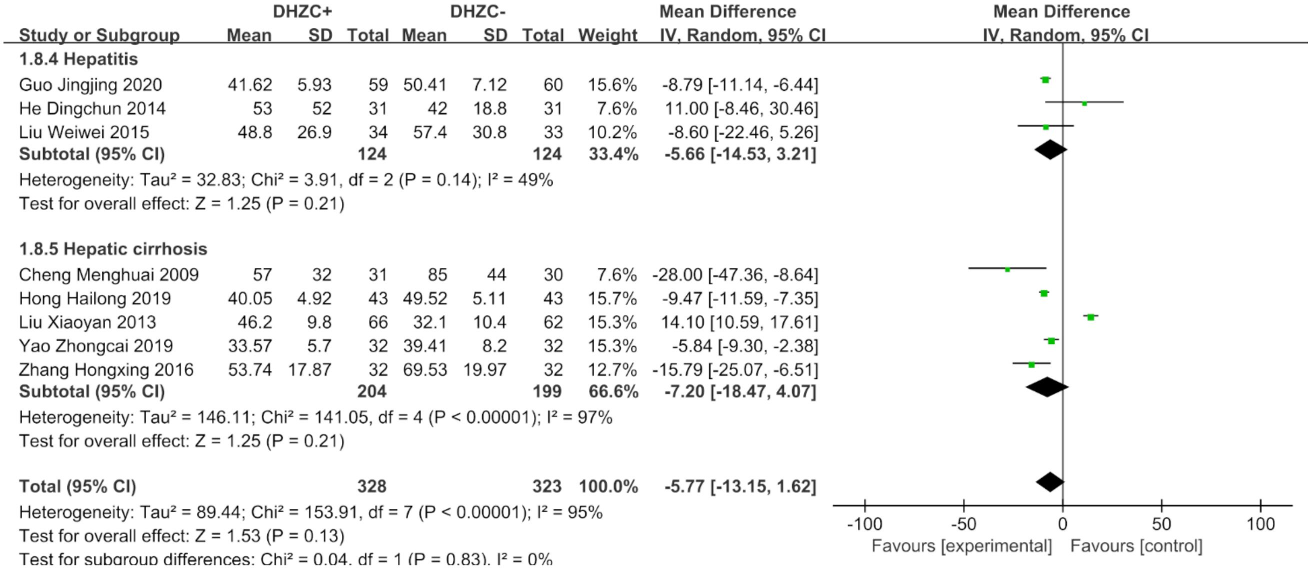

(B)

Study or Subgroup	DHZC+ Mean	DHZC+ SD	DHZC+ Total	DHZC- Mean	DHZC- SD	DHZC- Total	Weight	Mean Difference IV, Fixed, 95% CI
**1.6.4 Hepatitis**								
He Dingchun 2014	29.2	3	31	28.6	2.9	31	25.2%	0.60 [-0.87, 2.07]
Liu Weiwei 2015	31.9	3	34	29.7	2.7	33	29.1%	2.20 [0.83, 3.57]
**Subtotal (95% CI)**			**65**			**64**	**54.3%**	**1.46 [0.46, 2.46]**
Heterogeneity: Chi² = 2.44, df = 1 (P = 0.12); I² = 59%								
Test for overall effect: Z = 2.86 (P = 0.004)								
**1.6.5 Hepatic cirrhosis**								
Cheng Menghuai 2009	38	5	31	35	3	30	12.8%	3.00 [0.94, 5.06]
Liang Xiaoli 2009	36.9	2.2	30	35.3	4.8	30	15.2%	1.60 [-0.29, 3.49]
Liu Xiaoyan 2013	33.8	5.2	66	27.7	15.2	62	3.4%	6.10 [2.11, 10.09]
Pan Wen 2019	36.04	4.85	47	34.45	4.82	47	14.2%	1.59 [-0.36, 3.54]
**Subtotal (95% CI)**			**174**			**169**	**45.7%**	**2.33 [1.24, 3.42]**
Heterogeneity: Chi² = 4.97, df = 3 (P = 0.17); I² = 40%								
Test for overall effect: Z = 4.18 (P < 0.0001)								
**Total (95% CI)**			**239**			**233**	**100.0%**	**1.85 [1.12, 2.59]**
Heterogeneity: Chi² = 8.73, df = 5 (P = 0.12); I² = 43%								
Test for overall effect: Z = 4.93 (P < 0.00001)								
Test for subgroup differences: Chi² = 1.32, df = 1 (P = 0.25), I² = 24.3%								

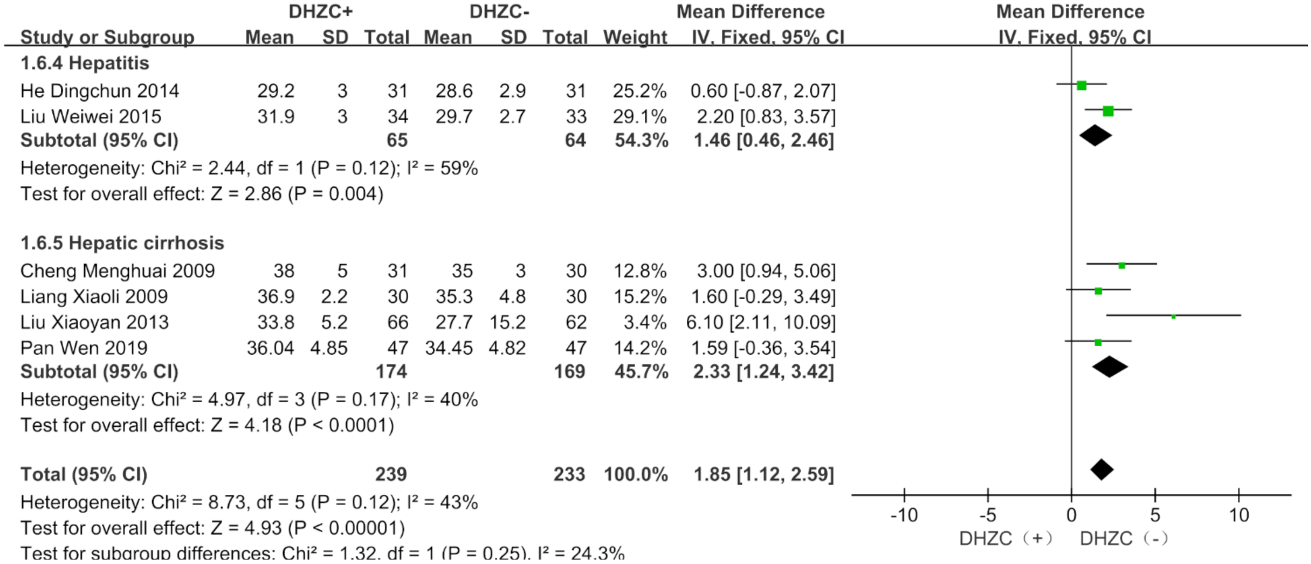

Supplementary Figure 9. The Effect of DHZCP based therapy on AST and ALB with a subgroup of stages of disease. (A) AST with a subgroup of stages of disease. Random effects models were applied. (B) ALB with a subgroup of stages of disease. Fixed effects models were applied.

(A)

Study or Subgroup	DHZC+ Mean	DHZC+ SD	DHZC+ Total	DHZC- Mean	DHZC- SD	DHZC- Total	Weight	Mean Difference IV, Random, 95% CI
**1.5.4 Hepatitis**								
Guo Jingjing 2020	20.35	3.04	59	25.6	3.95	60	14.7%	-5.25 [-6.52, -3.98]
He Dingchun 2014	65.4	23.2	31	72.5	19.8	31	4.9%	-7.10 [-17.84, 3.64]
Liu Weiwei 2015	24.5	6.8	34	21.4	5.7	33	13.0%	3.10 [0.10, 6.10]
Xu Qian 2006	11.4	6.3	45	12.8	7.9	45	13.1%	-1.40 [-4.35, 1.55]
**Subtotal (95% CI)**			**169**			**169**	**45.7%**	**-2.01 [-6.65, 2.64]**
Heterogeneity: Tau² = 17.49; Chi² = 28.20, df = 3 (P < 0.00001); I² = 89%								
Test for overall effect: Z = 0.85 (P = 0.40)								
**1.5.5 Hepatic cirrhosis**								
Cheng Menghuai 2009	28	13	31	41	14	30	8.3%	-13.00 [-19.79, -6.21]
Liang Xiaoli 2009	48.5	19.8	30	54	22.6	30	4.9%	-5.50 [-16.25, 5.25]
Liu Xiaoyan 2013	50.6	10.8	66	45.4	8.7	62	12.5%	5.20 [1.81, 8.59]
Pan Wen 2019	50.01	32.04	47	52.46	22.46	47	4.7%	-2.45 [-13.64, 8.74]
Yao Zhongcai 2019	18.25	2.98	32	21.09	3.45	32	14.5%	-2.84 [-4.42, -1.26]
Zhang Hongxing 2016	30.52	10.63	32	39.59	12.67	32	9.5%	-9.07 [-14.80, -3.34]
**Subtotal (95% CI)**			**238**			**233**	**54.3%**	**-4.21 [-9.38, 0.97]**
Heterogeneity: Tau² = 30.52; Chi² = 34.52, df = 5 (P < 0.00001); I² = 86%								
Test for overall effect: Z = 1.59 (P = 0.11)								
**Total (95% CI)**			**407**			**402**	**100.0%**	**-2.98 [-5.89, -0.08]**
Heterogeneity: Tau² = 14.54; Chi² = 64.99, df = 9 (P < 0.00001); I² = 86%								
Test for overall effect: Z = 2.01 (P = 0.04)								
Test for subgroup differences: Chi² = 0.38, df = 1 (P = 0.54), I² = 0%								

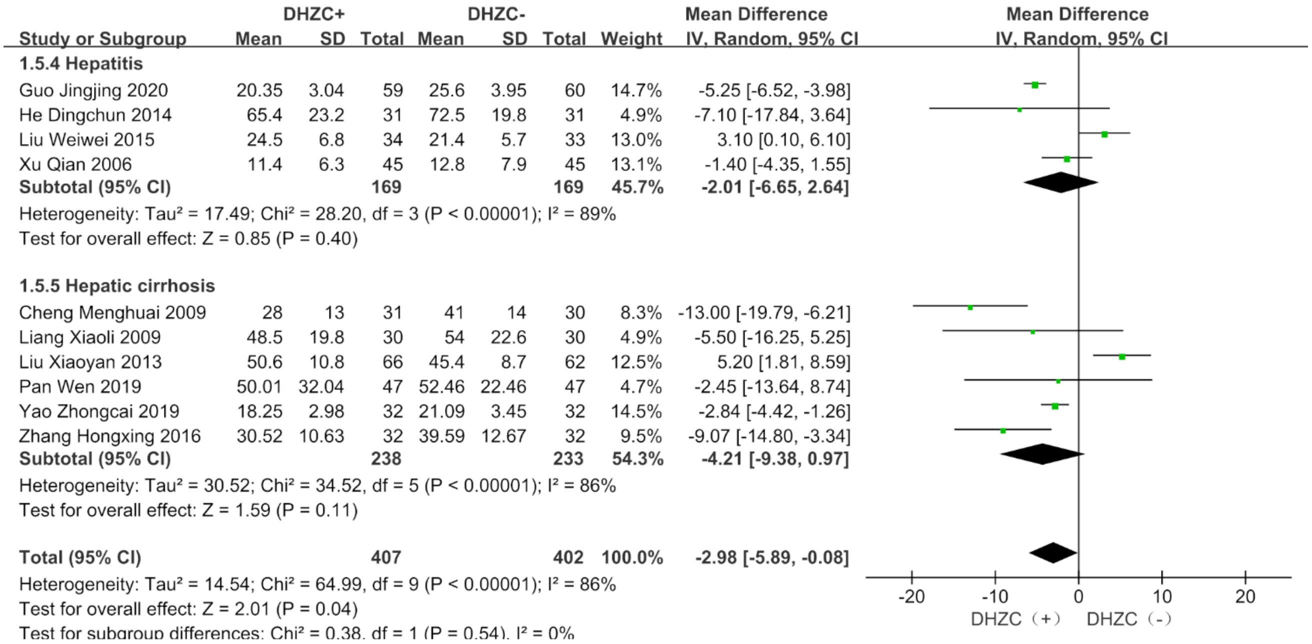

(B)

Study or Subgroup	DHZC+ Mean	DHZC+ SD	DHZC+ Total	DHZC- Mean	DHZC- SD	DHZC- Total	Weight	Mean Difference IV, Fixed, 95% CI
**1.6.1 Other Therapy**								
Cheng Menghuai 2009	38	5	31	35	3	30	12.8%	3.00 [0.94, 5.06]
Liang Xiaoli 2009	36.9	2.2	30	35.3	4.8	30	15.2%	1.60 [-0.29, 3.49]
Liu Weiwei 2015	31.9	3	34	29.7	2.7	33	29.1%	2.20 [0.83, 3.57]
Pan Wen 2019	36.04	4.85	47	34.45	4.82	47	14.2%	1.59 [-0.36, 3.54]
**Subtotal (95% CI)**			**142**			**140**	**71.4%**	**2.09 [1.22, 2.97]**
Heterogeneity: Chi² = 1.28, df = 3 (P = 0.73); I² = 0%								
Test for overall effect: Z = 4.70 (P < 0.00001)								
**1.6.5 Adefovir**								
He Dingchun 2014	29.2	3	31	28.6	2.9	31	25.2%	0.60 [-0.87, 2.07]
Liu Xiaoyan 2013	33.8	5.2	66	27.7	15.2	62	3.4%	6.10 [2.11, 10.09]
**Subtotal (95% CI)**			**97**			**93**	**28.6%**	**1.26 [-0.12, 2.64]**
Heterogeneity: Chi² = 6.44, df = 1 (P = 0.01); I² = 84%								
Test for overall effect: Z = 1.79 (P = 0.07)								
**Total (95% CI)**			**239**			**233**	**100.0%**	**1.85 [1.12, 2.59]**
Heterogeneity: Chi² = 8.73, df = 5 (P = 0.12); I² = 43%								
Test for overall effect: Z = 4.93 (P < 0.00001)								
Test for subgroup differences: Chi² = 1.01, df = 1 (P = 0.32), I² = 0.9%								

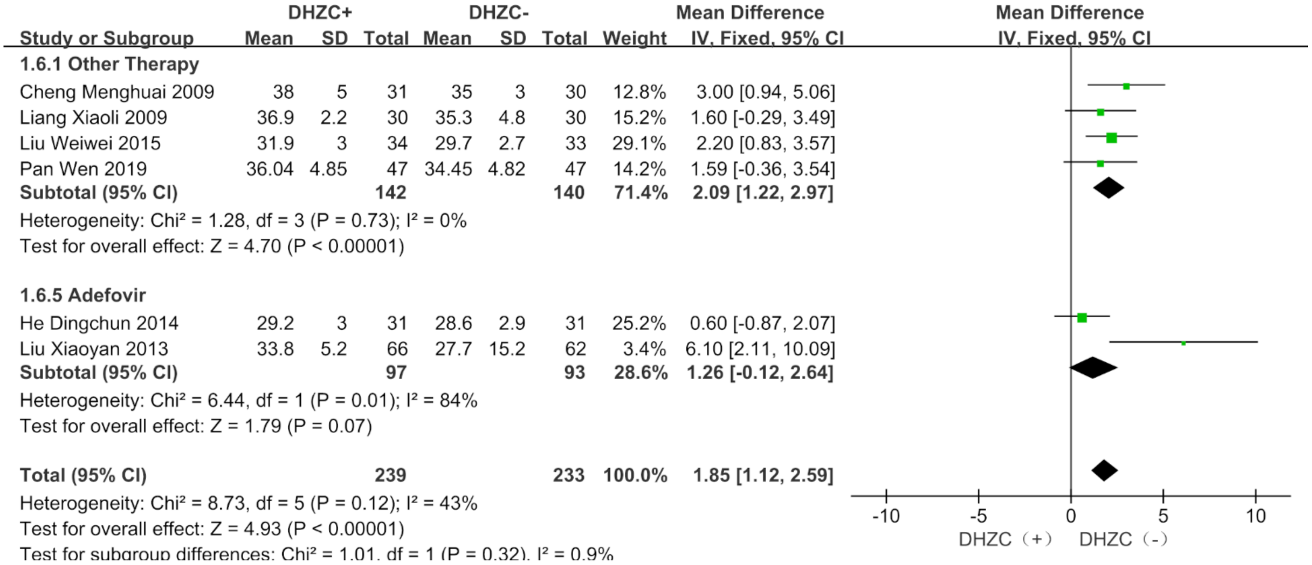

Supplementary Figure 10. The Effect of DHZCP based therapy on Tbil with a subgroup of stages of disease, and ALB with a subgroup of medication use. Random effects models were applied. (A) Tbil with a subgroup of stages of disease; (B) ALB with a subgroup of medication use.

Study or Subgroup	DHZC+ Mean	DHZC+ SD	DHZC+ Total	DHZC- Mean	DHZC- SD	DHZC- Total	Weight	Mean Difference IV, Random, 95% CI
**1.5.1 Other Therapy**								
Cheng Menghuai 2009	28	13	31	41	14	30	8.3%	-13.00 [-19.79, -6.21]
Guo Jingjing 2020	20.35	3.04	59	25.6	3.95	60	14.7%	-5.25 [-6.52, -3.98]
Liang Xiaoli 2009	48.5	19.8	30	54	22.6	30	4.9%	-5.50 [-16.25, 5.25]
Liu Weiwei 2015	24.5	6.8	34	21.4	5.7	33	13.0%	3.10 [0.10, 6.10]
Pan Wen 2019	50.01	32.04	47	52.46	22.46	47	4.7%	-2.45 [-13.64, 8.74]
Xu Qian 2006	11.4	6.3	45	12.8	7.9	45	13.1%	-1.40 [-4.35, 1.55]
**Subtotal (95% CI)**			**246**			**245**	**58.6%**	**-3.53 [-7.71, 0.64]**
Heterogeneity: Tau² = 18.63; Chi² = 35.09, df = 5 (P < 0.00001); I² = 86%								
Test for overall effect: Z = 1.66 (P = 0.10)								
**1.5.4 Entecavir**								
Yao Zhongcai 2019	18.25	2.98	32	21.09	3.45	32	14.5%	-2.84 [-4.42, -1.26]
Zhang Hongxing 2016	30.52	10.63	32	39.59	12.67	32	9.5%	-9.07 [-14.80, -3.34]
**Subtotal (95% CI)**			**64**			**64**	**24.0%**	**-5.32 [-11.30, 0.66]**
Heterogeneity: Tau² = 14.81; Chi² = 4.22, df = 1 (P = 0.04); I² = 76%								
Test for overall effect: Z = 1.74 (P = 0.08)								
**1.5.5 Adefovir**								
He Dingchun 2014	65.4	23.2	31	72.5	19.8	31	4.9%	-7.10 [-17.84, 3.64]
Liu Xiaoyan 2013	50.6	10.8	66	45.4	8.7	62	12.5%	5.20 [1.81, 8.59]
**Subtotal (95% CI)**			**97**			**93**	**17.5%**	**0.15 [-11.71, 12.01]**
Heterogeneity: Tau² = 59.15; Chi² = 4.58, df = 1 (P = 0.03); I² = 78%								
Test for overall effect: Z = 0.02 (P = 0.98)								
**Total (95% CI)**			**407**			**402**	**100.0%**	**-2.98 [-5.89, -0.08]**
Heterogeneity: Tau² = 14.54; Chi² = 64.99, df = 9 (P < 0.00001); I² = 86%								
Test for overall effect: Z = 2.01 (P = 0.04)								
Test for subgroup differences: Chi² = 0.69, df = 2 (P = 0.71), I² = 0%								

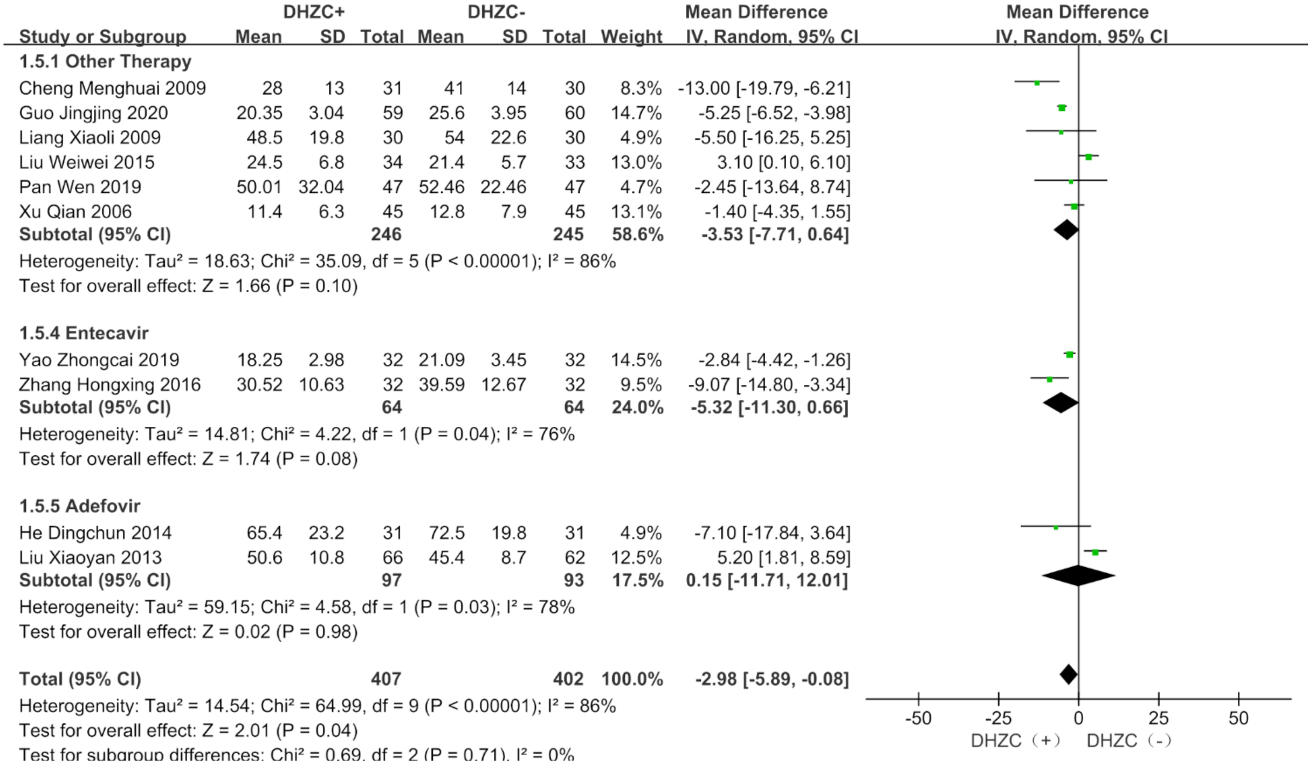

Supplementary Figure 11. The Effect of DHZCP based therapy on Tbil with a subgroup of medication use. Random effects models were applied.
